# RNA modifications detection by comparative Nanopore direct RNA sequencing

**DOI:** 10.1038/s41467-021-27393-3

**Published:** 2021-12-10

**Authors:** Adrien Leger, Paulo P. Amaral, Luca Pandolfini, Charlotte Capitanchik, Federica Capraro, Valentina Miano, Valentina Migliori, Patrick Toolan-Kerr, Theodora Sideri, Anton J. Enright, Konstantinos Tzelepis, Folkert J. van Werven, Nicholas M. Luscombe, Isaia Barbieri, Jernej Ule, Tomas Fitzgerald, Ewan Birney, Tommaso Leonardi, Tony Kouzarides

**Affiliations:** 1 European Molecular Biology Laboratory, European Bioinformatics Institute, Wellcome Genome Campus, Hinxton, Cambridge, UK; 2 The Gurdon Institute, University of Cambridge, Tennis Court Road, Cambridge, UK; 3 The Milner Therapeutics Institute, Jeffrey Cheah Biomedical Centre, University of Cambridge, Puddicombe Way, Cambridge, UK; 4 INSPER - Institute of Education and Research, São Paulo, SP Brazil; 5 Istituto Italiano di Tecnologia (IIT), Center for Human Technologies (CHT), Genova, Italy; 6 The Francis Crick Institute, London, UK; 7 Department of Neuromuscular Diseases, UCL Queen Square Institute of Neurology, Queen Square, London, UK; 8 Department of Pathology, Division of Cellular and Molecular Pathology, University of Cambridge, Cambridge, UK; 9 Department of Pathology, University of Cambridge, Tennis Court Road, Cambridge, UK; 10 Department of Genetics, Environment and Evolution, UCL Genetics Institute, London, UK; 11 Okinawa Institute of Science & Technology Graduate University, Okinawa, Japan; 12 Center for Genomic Science of IIT@SEMM, Istituto Italiano di Tecnologia (IIT), Milan, Italy; 13 Present Address: Oxford Nanopore Technologies, Gosling Building, Oxford Science Park, Oxford, UK

**Keywords:** Bioinformatics, Transcriptomics, Software, RNA sequencing

## Abstract

RNA molecules undergo a vast array of chemical post-transcriptional modifications (PTMs) that can affect their structure and interaction properties. In recent years, a growing number of PTMs have been successfully mapped to the transcriptome using experimental approaches relying on high-throughput sequencing. Oxford Nanopore direct-RNA sequencing has been shown to be sensitive to RNA modifications. We developed and validated Nanocompore, a robust analytical framework that identifies modifications from these data. Our strategy compares an RNA sample of interest against a non-modified control sample, not requiring a training set and allowing the use of replicates. We show that Nanocompore can detect different RNA modifications with position accuracy in vitro, and we apply it to profile m^6^A in vivo in yeast and human RNAs, as well as in targeted non-coding RNAs. We confirm our results with orthogonal methods and provide novel insights on the co-occurrence of multiple modified residues on individual RNA molecules.

## Abstract (web summary)

Nanopore direct RNA Sequencing data contain information about the presence of RNA modifications, but their detection poses substantial challenges. Here the authors introduce Nanocompore, a new methodology for modification detection from Nanopore data.

## Introduction

RNA post-transcriptional modifications (PTMs) are a pervasive feature common to all domains of life. They arise from covalent alteration or isomerisation of nucleotides, typically involving the addition of chemical groups to different positions of the nitrogenous bases or the ribose cycle. To date, over 150 modifications have been found throughout all classes of RNAs, with the most common modification being methylation^1^. PTMs are deposited and catalytically removed by specific enzymes and can be recognized by specific ‘reader’ proteins. Overall, PTMs influence fundamental properties and functions of RNAs, including their stability, structure, intermolecular interactions and cellular localization^2,3^.

N6-Methyladenosine (m6A) is the best characterised PTM and the most abundant in mRNAs and long non-coding RNAs (lncRNAs). It is deposited mainly by the METTL3/METTL14/WTAP complex and has a variety of functions such as regulation of nuclear export, translation, and degradation of RNAs^4,5^. Other modifications, including Inosine (I), 5-methylcytosine (m5C), pseudouridine (Ψ) N6,N6-dimethyladenosine (m6,2A), 1-methylguanosine (m1G), 2′-O methyladenosine (2′-OMeA), and 7-methylguanosine (m7G), are increasingly recognized as important for the regulation of different RNAs in physiological and pathological contexts, including cancer^6,7^.

The majority of current methods for mapping PTMs rely on RNA immunoprecipitation, chemoselective alteration, or specific signatures resulting from reverse transcription (RT), and despite being the current gold standard have certain limitations, such as (1) the need to develop ad hoc protocols for each PTM, (2) cross reactivity or low sensitivity of antibodies or chemical reactions, and (3) biases induced by the complex multi-step experimental protocols^8,9^.

The recent advances in Nanopore direct RNA sequencing (DRS) have allowed, for the first time, direct sequencing of full-length native RNA molecules without the need for RT or amplification. Importantly, a number of studies have shown that DRS data intrinsically contain information about RNA modifications^10–12^. In Nanopore DRS, a single RNA molecule is ratcheted by a molecular motor through a protein pore embedded in a synthetic membrane. The passage of nucleobases through the narrowest section of the pore (reader-head) alters the flow of ions across the membrane, depending on the chemical composition of the bases. At any given point in time, approximately 5 nucleotides (commonly referred to as a *kmer*) reside within the reader-head of R9 pores, leading to a strong kmer specific signal alteration. Crucially, the presence of nucleotide modifications can induce discernible shifts in current intensity and in the time the nucleic acid sequence resides inside the pore (dwell time)^7,10^.

In recent years, the scientific community has devoted substantial resources toward the development of experimental and analytical strategies for the detection of RNA modifications. These efforts have generated a number of algorithms and software packages, which have been extensively reviewed elsewhere^13^. The current approaches for modification detection based on Nanopore data can be divided into two categories: those based on the detection of modification-induced basecalling errors and those based on the analysis of the electrical signal. The first strategy, which is implemented in tools such as Epinano^14^, DiffErr^15^, Eligos^16^, and Drummer^17^, has shown interesting results despite not considering the effects of RNA modification on the raw electrical signal; however, modern basecalling models tend to become more insensitive to common PTM, with the risk that methods of this group could quickly become ineffective at detecting modifications. On the other hand, methods based on raw signal space analyses (such as Tombo^18^, Mines^19^, xPore^20^, nanom6A^21^, nanoRMS^22^, nanoDoc^23^, Yanocomp^24^, and Penguin^25^) can lead to richer comparative analyses, but are more complicated and come with steeper computational costs. The methods described above can be further classified into two groups: de novo detection methods, that use a trained model to identify modifications, and comparative methods, where differences between two samples are evaluated to infer the presence of a modification. At present, de novo strategies are often hindered by the difficulty to generate a training set containing all kmer contexts with and without modifications. For this reason, the majority of existing methods instead undertake a comparative approach, where the sample of interest is compared to a reference sample devoid of modifications. Here we introduce Nanocompore, a flexible and versatile analysis method dedicated to the detection of RNA modifications from DRS datasets in signal space. To identify potential modification sites, Nanocompore uses a model-free comparative approach based on a 2 components Gaussian mixture model, where an experimental RNA sample is compared against a sample with fewer or no modifications. Potentially, this can be applied to any modification, provided that an appropriate control depleted of the modification is available, and that the modification significantly alters the current signal. We demonstrate this for seven different RNA modifications in synthetic oligonucleotides, as well as extensively for m6A in coding and noncoding native RNAs in yeast and mammalian cells. Nanocompore includes several unique features: (1) robust signal realignment based on Nanopolish, (2) modelling of the biological variability, (3) ability to run multiple statistical tests, (4) prediction of RNA modifications using both signal intensity and duration (dwell time), and (5) availability of an automated pipeline that runs all the preprocessing steps. Finally, the results generated by Nanocompore can also be leveraged to infer RNA modifications at single molecule resolution.

## Results

### Nanocompore data preparation and statistical basis

**Fig. 1:**
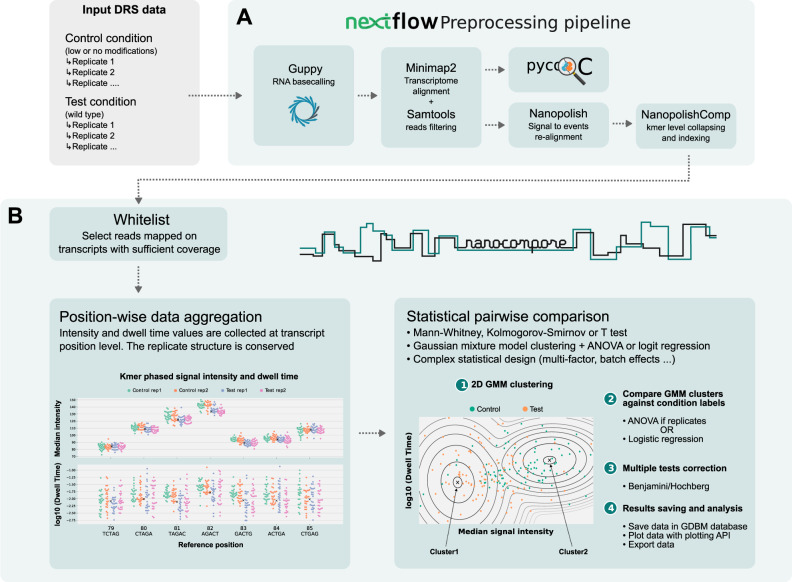
Overview of data preparation and Nanocompore steps. **A** Raw fast5 reads from 2 conditions are basecalled with Guppy, filtered with Samtools and the signal is then resquiggled with Nanopolish eventalign. The output of Nanopolish is then collapsed and indexed at the kmer level by NanopolishComp Eventalign_collapse. **B** Nanocompore aggregates median intensity and dwell time at transcript position level. The data is compared in a pairwise fashion position per position using univariate tests (KS, MW, t-tests) and/or a bivariate GMM classification method. The p-values are corrected for multiple tests and these data are saved in a database for further analyses. The signal graph is as an illustration not representative of all possible kmers.

Nanocompore detects potential RNA modifications by comparing DRS datasets from one experimental test condition containing specific RNA modifications to one control condition containing significantly fewer or no modifications. Ideally, the control RNA is isolated from a cell harbouring either a knock-down (KD) or a knock-out (KO) of a gene encoding an RNA modifying enzyme. Alternatively, for small scale comparison, it is also possible to use either an in vitro transcribed or synthetic RNA containing canonical RNA bases only. We have developed an automated Nextflow pipeline (https://github.com/tleonardi/nanocompore_pipeline) that automatically runs the entire analysis from preprocessing of raw Nanopore data (Fig. 1A), to modified-base detection with Nanocompore (https://github.com/tleonardi/nanocompore, Fig. 1B). Firstly, reads are grouped by reference transcript and transcripts with coverage above a user-specified threshold are used for subsequent analyses. Then, two parameters - the median signal intensity and the log10(dwell time)—are collected from each read and aggregated at the transcript position level. The aggregated data are compared in a pairwise fashion, one position at the time. For the identification of modified positions, Nanocompore supports robust univariate pairwise tests on current intensity or dwell time (Kolmogorov-Smirnov test, KS). In addition, we implemented a more advanced bivariate classification method based on 2 components Gaussian mixture model (GMM) clustering followed by a logistic regression test (logit) to determine if there is a significant difference in the distribution of reads into the two clusters between conditions. Furthermore, we and others observed that DNA and RNA modifications can have an intrinsic effect on the local signal upstream or downstream of the modification position. Thus, to evaluate the effect of modifications on the proximal sequence context, Nanocompore offers the option to use Hou’s method to combine the non-independent *p*-values of neighbouring kmers (see Materials and Methods)^26^. The *p*-values are then corrected for multiple tests using Benjamini-Hochberg’s procedure^27^ and the results are stored in a lightweight database. Users can obtain a tabular text dump of the database or use the extensive Nanocompore API to explore the results and generate ready-to-publish plots.

### In silico and in vitro validation

We first tested Nanocompore on in silico data that simulated the presence of RNA modifications. This technical control confirmed Nanocompore’s capacity to detect alterations in current intensity and/or dwell time between two samples (see Supplementary Information and Fig S1, S2).

**Fig. 2:**
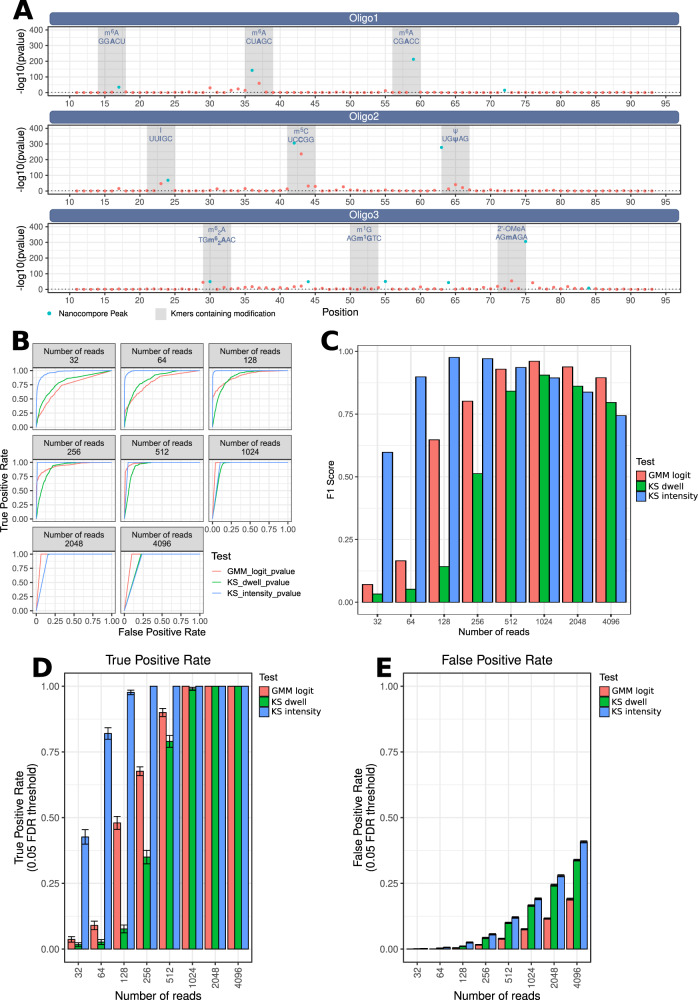
Nanocompore benchmarks with synthetic modified oligonucleotides. **A** Nanocompore *p*-values (GMM logit method, y-axis) reported at each position (x-axis) along three oligonucleotides of 100nt carrying multiple modifications at defined positions. Oligo1: three m6A sites in different sequence contexts; Oligo2: I, m5C and Ψ; Oligo3: m6,2A, m1G and 2’-OMeA Kmers shown in blue represent the peaks identified through Nanocompore’s peak calling procedure. Shaded areas contain the 5 consecutive kmers that contain each modification. Each oligonucleotide was sequenced in a separate flowcell, producing on average 648,543.5 reads after quality filtering. The dotted horizontal lines correspond to a p-value of 0.01. **B** Nanocompore ROC curves for m6A detection (Oligo1) at varying levels of coverage and using different statistical tests (GMM logit test, KS test on intensity or KS test on dwell time). **C** F1 score for m6A detection (Oligo1) with the GMM logit test, KS test on intensity or KS test on dwell time at varying levels of coverage. Nominal *p*-value threshold of 0.05. **D**, **E** True Positive (**D**) and False Positive (**E**) rates for m6A detection (Oligo1). The values reported are the means of *n* = 100 artificial samples generated as described (see Materials and Methods). The error bars show the 95% confidence interval. TPR and FPR were calculated at a nominal *p*-value threshold of 0.05.

To further validate the ability of Nanocompore to detect RNA modifications in real Nanopore data, we designed 3 oligonucleotides carrying multiple modifications including m6A in three different sequence contexts, I, m5C, Ψ, m6,2A, m1G, and 2’-OMeA (see Materials and Methods). The data generated from the modified oligos was then analysed with Nanocompore using an unmodified oligo as the reference condition. These results show that Nanocompore can detect all modifications tested (Figs. 2A and S3), including the m6A modification both in the canonical DRACH motif and non-DRACH sequence contexts^28^. Of all modifications tested, m1G was the only one that instead of being detected in one of the modification-containing kmers gave a significant signal peak 1 kmer downstream. However, also for the other modifications we observed that the intensity shift at modified sites spreads to adjacent kmers containing the m6A residue (Fig. S3). This shows that a modification can alter the signal locally and supports the rationale of combining the p-values of neighbouring kmers.

To better gauge the accuracy of Nanocompore at coverage levels representative of real experiments, we generated 100 subsampled datasets containing random samples of 32 to 4096 reads, doubling at each step. By analysing such datasets with Nanocompore, we observed that the GMM-logit method had lower sensitivity but higher specificity than the non-parametric tests on intensity or dwell time (Fig. 2B). This was also reflected in the GMM-logit test having the best F1 score at coverage greater than 512 reads (Fig. 2C–E). Overall, at a *p*-value cutoff of 0.05 and 512 reads coverage, the GMM-logit test had a mean accuracy of 94.48% at detecting m6A and 89.8% at detecting other modifications.

**Fig. 3:**
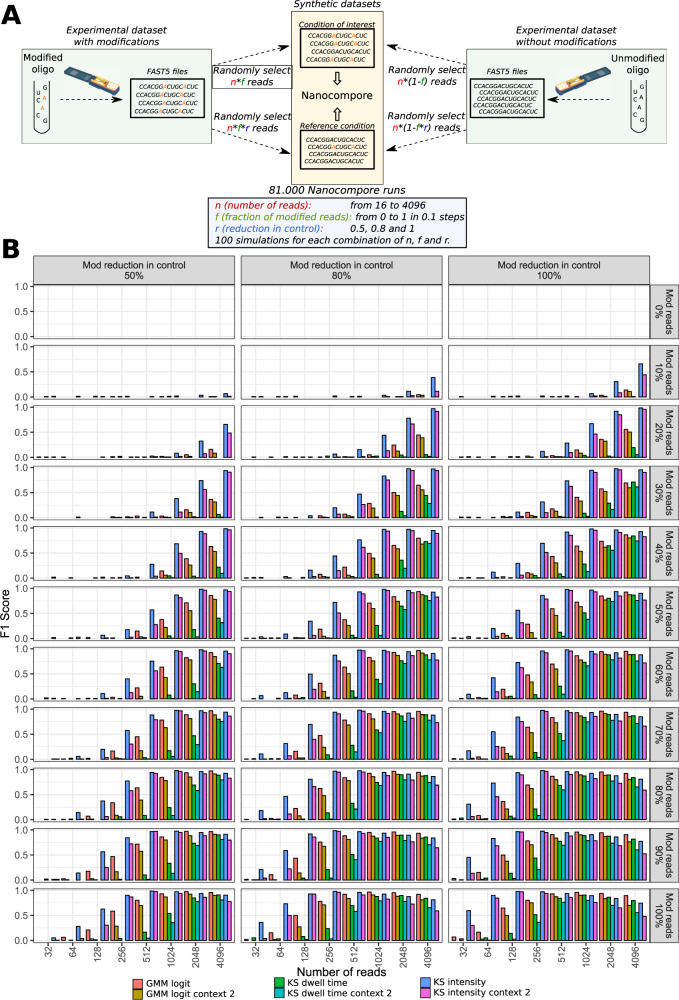
Nanocompore benchmarks with simulated modification stoichiometry and knock down efficiency. **A** Diagram illustrating the procedure used to generate in silico datasets at varying levels of coverage, modification stoichiometry and knock down efficiency. **B** Plots showing the F1 score for m6A detection (Oligo1) with the various tests implemented in Nanocompore at varying levels of (1) coverage (x-axis), (2) modification reduction in control (columns), and (3) percentage of modified reads (rows). Nominal *p*-value threshold of 0.05.

We then reasoned that the results obtained with these modified oligos are only representative of the extreme situation where 100% of the RNA is modified in the condition under study whereas the modification is completely absent from the control condition. In order to evaluate the performance of our method under conditions more representative of real experimental scenarios, we generated in silico datasets by mixing known proportions of modified and unmodified reads. Such datasets where generated for each intersection of 3 possible factors: (1) % of modified reads in experimental condition (ranging from 0% to 100% in steps of 10%, effectively simulating modification stoichiometry); (2) % of modification reduction in control condition (100%, 80% or 50% reduction, effectively simulating knock-down efficiency), and (3) read coverage (from 16 to 4096 reads per dataset). For each combination of these three factors we generated 100 independent datasets that were then analysed with Nanocompore, for a total of 80,000 runs (Fig. 3A). By knowing the ground-truth modification state in each run we could measure accuracy and produce ROC curves for all conditions tested (Fig S4, S5, S6). As expected, we observed that the accuracy varied greatly according to the coverage as well as to the relative fraction of modified reads in the test and control conditions (Fig S7). For example, at coverage levels below 128 reads we found that Nanocompore could hardly detect modified sites unless the modification stoichiometry and/or knock-down efficiency were high. On the other hand, at a coverage of 4096 reads, we could detect 75% of m6A sites when as little as 20% of the reads are modified (Fig. S7).

These simulations also allowed us to better investigate the performance of the different tests implemented in Nanocompore. We observed that the KS tests on current intensity or dwell time achieved the highest sensitivity at the cost of lower specificity, in particular at high levels of coverage. On the other hand, the GMM logit test has the lowest False Positive Rate overall and the best balance between precision and sensitivity (i.e. highest F1 score) at high coverage (Fig. 3B, S6 and S7). Additionally, our data show that different modifications and/or different sequence contexts have heterogeneous effects on the current intensity and/or dwell time of Nanopore data (Fig. S3), and the GMM test is the only one that simultaneously captures both. Therefore the GMM-logit test is the most suitable choice to analyse RNA modifications in complex transcriptomes, where the sequencing coverage is heterogeneous between transcripts and where the effect of the modification on current and dwell time is not known. For all these reasons, all the analysis in this article will make use of the GMM-logit test unless otherwise stated.

As a further control for Nanocompore sensitivity, we re-analysed DRS dataset of 16S rRNA from *Escherichia coli* strain MRE600 knock-out for *RsmG* or *RsuA*, which are responsible for an m7G residue at position G527 and Ψ at position 516 respectively^12^. In both cases, Nanocompore was able to detect the modified nucleotides as highly significant (Fig. S8, *p*-value<10^−300^ for both sites).

### Benchmarking nanocompore with metacompore

Having validated the accuracy of Nanocompore on simulated and synthetic data, we sought to compare the in vivo performance of Nanocompore with that of other methods based on Nanopore sequencing. We first focused on the m6A modification in yeast, a species with a relatively small transcriptome and with a comprehensive annotation of known m6A sites based on techniques orthogonal to Nanopore sequencing. We generated a *Saccharomyces cerevisiae* strain KO for *IME4* (*ime4Δ*), the only known m6A methyltransferase in yeast. We then used DRS to sequence the polyA+ transcriptome in Wild Type (WT) cells as well as *ime4Δ* cells. We sequenced three biological replicates per condition in individual flowcells, generating a total of 14,554,547 reads and obtaining a coverage above 30x for 2,523 genes (40% of the total annotated transcriptome). Nanocompore analysis of such a dataset identified 15,961 significant kmers in 1,510 distinct transcripts (FDR 1%, Fig. S9A). Since a single modification can affect the signal of multiple neighbouring kmers, we refined our predictions with a peak calling algorithm, finding 10,217 peaks with a median of 3 peaks per transcript. In line with current knowledge on m6A, we found that Nanocompore peaks were enriched in proximity to the stop codon of mRNAs (Fig. S9B) and were also enriched for the canonical DRACH motif (Fig. S9C). To assess the accuracy of Nanocompore’s results we measured the overlap between the predicted m6A sites identified and known m6A sites annotated in an orthogonal reference set of yeast m6A sites^29,30^ (see Materials and Methods). This analysis revealed that 21% (124/602) of known m6A sites overlap with a Nanocompore peak, whereas 8% (124/1549) of the sites identified by Nanocompore were also supported by a peak in the orthogonal reference set (Fig. S9D, E).

In order to compare our results with those obtained through other tools, we developed Metacompore, a software pipeline written in the Snakemake language^31^ that automatically runs 6 different algorithms for modification detection, namely: Nanocompore, Tombo, Eligos, Diff_err, Epinano and MINES (see **Materials and Methods and** Supplementary Table 1 for a comparison of their features). We then used the collection of m6A sites in the orthogonal reference set as a ground truth, and used it to calculate the sensitivity, specificity and precision of each method. Since Epinano and MINES are designed to only detect m6A sites within the DRACH motif, we performed two separate analyses, one that considered all kmers but excluded Epinano and MINES and another one that only considered DRACH kmers and included Epinano and MINES.

When considering all kmers, we found that Eligos2 had the highest sensitivity (45.8%) of all methods tested, while Nanocompore’s GMM method and GMM context 2 method had a sensitivity of only 16% and 5.5% respectively (Fig. S10A, nominal FDR threshold 1%, log odds ratio threshold 0.5). On the other hand, Nanocompore had the highest specificity of all methods tested (98.3% and 99.7% for GMM and GMM context 2 respectively) whereas Tombo had the lowest (26.8%, Fig. S10B). We then used the F1 score to measure the balance between sensitivity and specificity, finding that Nanocompore achieved the best overall score (0.0994, Fig. S10C) closely followed by diff_err (0.0969). Similarly, in terms of precision (fraction of True Positive m6A sites out of all sites predicted as m6A) Nanocompore GMM context 2 achieved the best result (Fig. S10D), with an 1.8-fold increase over the second most precise method diff_err (F1 scores of 0.153 and 0.084 for Nanocompore and diff_err, respectively).

We then repeated a similar analysis only considering DRACH kmers but including Epinano and MINES in the comparisons. This time we found that Eligos achieved the best balance of sensitivity and specificity with an F1 Score of 0.287, whereas Nanocompore had the second best score of 0.180 (Fig. S10E–G). However, also in this case Nanocompore GMM context 2 achieved highest precision at the cost of lower sensitivity, with 43.8% of its predicted m6A sites being confirmed by the orthogonal reference set (Fig. S10H).

Nanocompore, similarly to Eligos and diff_err, also reports the odds ratio of modified sites, which indicates the magnitude of the effect (see Materials and Methods). We therefore also measured the sensitivity and specificity of Nanocompore at a stringent log odds ratio threshold. As expected, we found that more stringent filtering increased specificity at the cost of lower sensitivity, with an overall increase in precision (Figure S10I–L). Finally, we also found that the KS tests on intensity or dwell time alone had worse performance compared to GMM both in terms of F1 score and precision, further supporting our approach of combining intensity and dwell time through Gaussian Mixture Modeling.

### Transcriptome-wide m6A profiling in mammalian cells

**Fig. 4:**
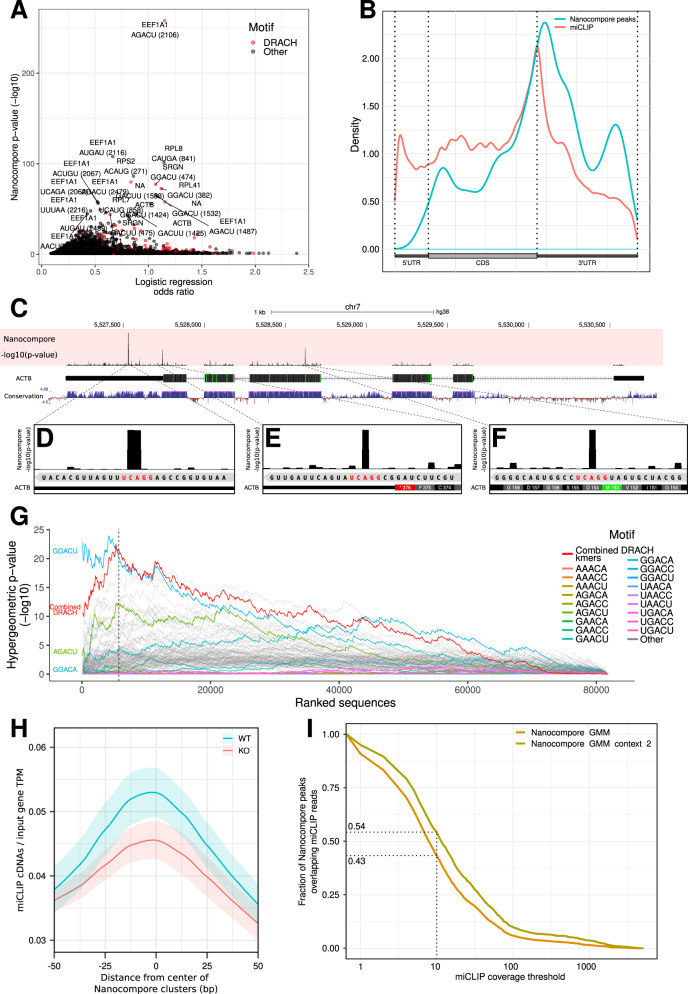
m6A profiling in MOLM13 cells. **A** Sharkfin plot showing the absolute value of the Nanocompore logistic regression log odd ratio (GMM logit method with context 2, x-axis) plotted against its *p*-value (-log10, y-axis, see Material and Methods). Each point represents a specific kmer of a transcript. Red points are DRACH kmers. **B** Metagene plot showing the distribution of significant m6A sites identified by Nanocompore (blue) and miCLIP (red). **C** Genome browser screenshot showing METTL3-dependent m6A sites in the ACTB transcript. The *p*-value track reports the Nanocompore GMM+Logistic regression method (see Material and Methods). **D–F** As in C but showing the three most significant β-actin sites at higher magnification. The sequence reported at the bottom corresponds to the RNA sequence in the 3’ to 5’ orientation, as the ACTB transcript is encoded on the minus strand. The m6A consensus GGACU sequences are highlighted in red. **G** Sylamer plot showing kmer enrichment in Nanocompore significant sites. The x-axis reports all Nanocompore sites with *p*-value<0.5 ranked from the most to the least significant. The y-axis reports the uncorrected Sylamer hypergeometric p-value of enrichment (one-sided test) of a certain motif in the first x Nanocompore sites vs the rest. The vertical dotted line delineates Nanocompore sites with *p*-value<0.01 (to the left of the line). The red line corresponds to the combined *p*-value (Fisher’s method) of all DRACH kmers. **H** m6A miCLIP coverage of clusters of significant Nanocompore sites (GMM logit (context 2) *p*-value<0.01). The y-axis shows the mean input-normalised miCLIP counts across sites. Shaded regions on the plot represent the mean±the standard deviation at each position in the profile (WT miCLIP *n* = 4, KO *n* = 2). Both the mean and bounds were smoothed using loess regression with a span of 0.6. The difference between WT and KO in the windows 0+/-20nt is statistically significant (*p*-value = 7.90 × 10^−11^, Mann-Whitney test). **I** Plot showing the fraction of Nanocompore significant peaks supported by a varying number of miCLIP reads (x-axis) in WT MOLM13 cells.

We then sought to study the m6A modification in mammalian cells, where METTL3-METTL14 heterodimers form a N6-methyltransferase complex that methylates adenosine residues at the N(6) position of specific RNAs. Since m6A is required for development and maintenance of acute myeloid leukemia^32,33^, it is of particular importance to accurately map it in leukemia cells. We therefore used DRS to profile the poly-A^+^ transcriptome of human MOLM13 cells with inducible shRNA-mediated knock-down (KD) of METTL3, as well as control Wild Type (WT) MOLM13 transfected with a scrambled shRNA. We sequenced RNA from two biological replicates per condition on independent Minion flow cells after 4 days of induced KD of METTL3, yielding a total of 3,768,380 reads. After applying a 30× coverage threshold, we obtained data for 751 unique transcripts robustly expressed in all samples (Fig. S11A–C). Overall, we observed a high correlation of expression levels between samples showing the consistency of the datasets (*R*^2^ of 0.969, Fig. S11D–F). We then used Nanocompore to map the location of METTL3-dependent m6A sites in human transcripts from MOLM13 cells and found 11,995 significant kmers (FDR 1%), corresponding to 1570 peaks in 216 transcripts, with a median of 3 peaks per transcripts (Fig. 4A, Fig. S12). As an example, we found 40 peaks (337 kmers with *p*-value<0.01, Fig. 4C) in the β-actin (ACTB, ENST00000646664) mRNA. Interestingly, the 3 most significant β-actin hits are “GGACU” kmers, perfectly matching the canonical m6A DRACH motif (Fig. 4C–F). On a transcriptome-wide scale, we reproduced previous observations showing that METTL3-dependent m6A sites are enriched in the immediate vicinity of mRNA stop-codons (Fig. 4B)^4,34^. Additionally, we used Sylamer^35^ to identify enriched kmers in the Nanocompore significant kmers, finding a 4.3 fold enrichment for the consensus GGACU motif in the Nanocompore sites with *p*-value<0.01 (hypergeometric *p*-value = 4.3 × 10^-21^, Fig. 4G). Lastly, we generated miCLIP datasets from MOLM13 cells targeted with METTL3 CRISPR gRNAs to compare the results obtained with Nanocompore with an orthogonal high-resolution method. We found that 54% of Nanocompore sites were supported by miCLIP in WT cells (Fig. 4H, I) and Nanocompore positive sites also showed a significant reduction of miCLIP crosslink sites upon METTL3 KO (*p*-value = 7.90 × 10^−11^, Mann–Whitney test, Fig. 4H and Fig. S13). Overall, these results show that Nanocompore is capable of identifying enzyme-specific RNA modifications transcriptome-wide and that these findings are in agreement with previous techniques.

**Fig. 5:**
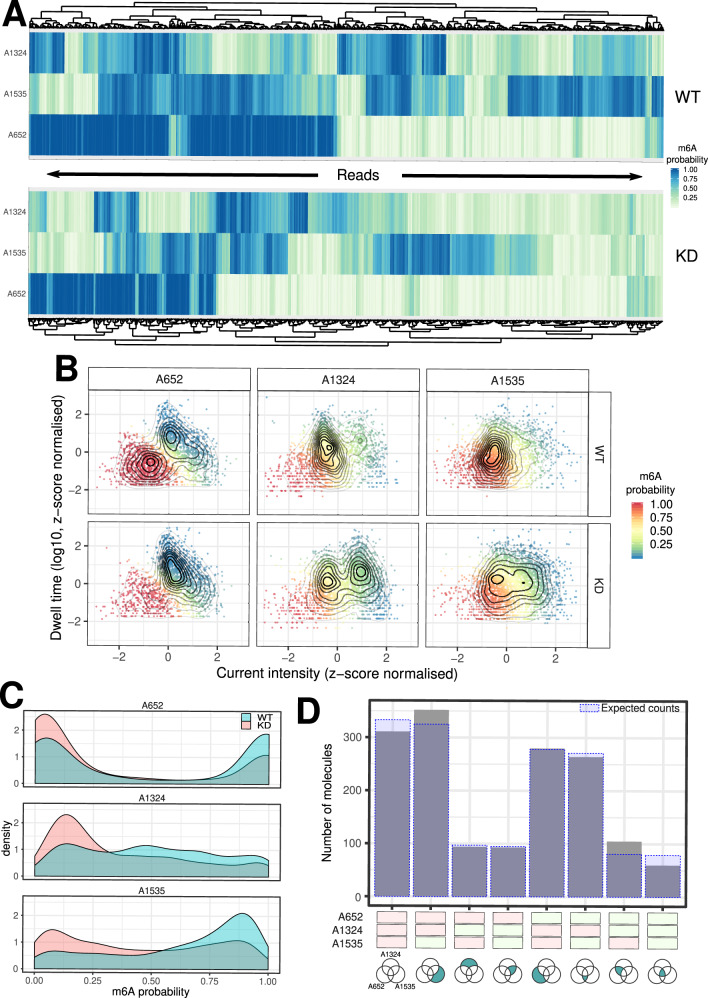
Single molecule identification of m6A sites. **A** Heatmap organised by hierarchical clustering showing the probability of A652, A1324, and A1535 in the β-actin gene to be modified in the WT and METTL3 KD samples. Each column corresponds to a single molecule. **B** Scatter plot with overlaid kernel density estimates showing the scaled median intensity vs the scaled log10 dwell time for each read covering A652, A1324 and A1535. Data points are colour coded according to the probability that the read belongs to the cluster of m6A modified reads. For visualisation purposes the x- and y-axis were limited to the +/-3 range. **C** Density plot showing the distribution of modification probability for A652, A1324, and A1535 of β-actin in WT (blue) and KD (red). **D** Bar chart showing the number of molecules identified in each of the 8 possible m6A configurations for the A652, A1324, and A1535 sites of β-actin. Each site was considered modified if the modification probability was >0.75. The shaded blue areas indicate the expected number of molecules in each given configuration under the null hypothesis of independence of the three modifications.

The identification of RNA modifications outlined so far operates at consensus level, i.e., looking at the distribution of signal across the entire population of reads. However, the information obtained from GMM clustering at the population level can be leveraged to calculate the probability of each read to belong to the modified or unmodified cluster. Hence, it is possible to assign modification probabilities at the single-molecule, single-site level. As a proof of concept, we calculated the single-molecule modification probabilities of the three β-actin high-confidence m6A sites previously described (Fig. 4C–F). We found that these three sites are methylated at different degrees: 45% of β-actin molecules methylated with high-confidence (probability >0.75) at position A652, 23% at position A1324 and 49% at position A1535. As expected, we also found that the fraction of methylated molecules decreased at all three sites in the METTL3 KD condition (26%, 14%, and 27% of molecules methylated at A652, A1324 and A1535 respectively, Fig. 5A–C). We further asked whether the presence of an m6A modification at one of these three sites influences the probability that the same molecule is modified at the other sites. Taking into account the underlying frequency of modification at each site, we calculated the conditional probabilities for all possible combinations of 0, 1, 2, or 3 modifications to co-occur in the same molecule (Fig. 5D). This analysis showed that the observed and expected modification frequencies do not differ significantly, suggesting that methylation of these three sites are independent events (*p*-value = 0.4, see Materials and Methods).

### Modification mapping in snRNA 7SK by high coverage targeted sequencing

**Fig. 6:**
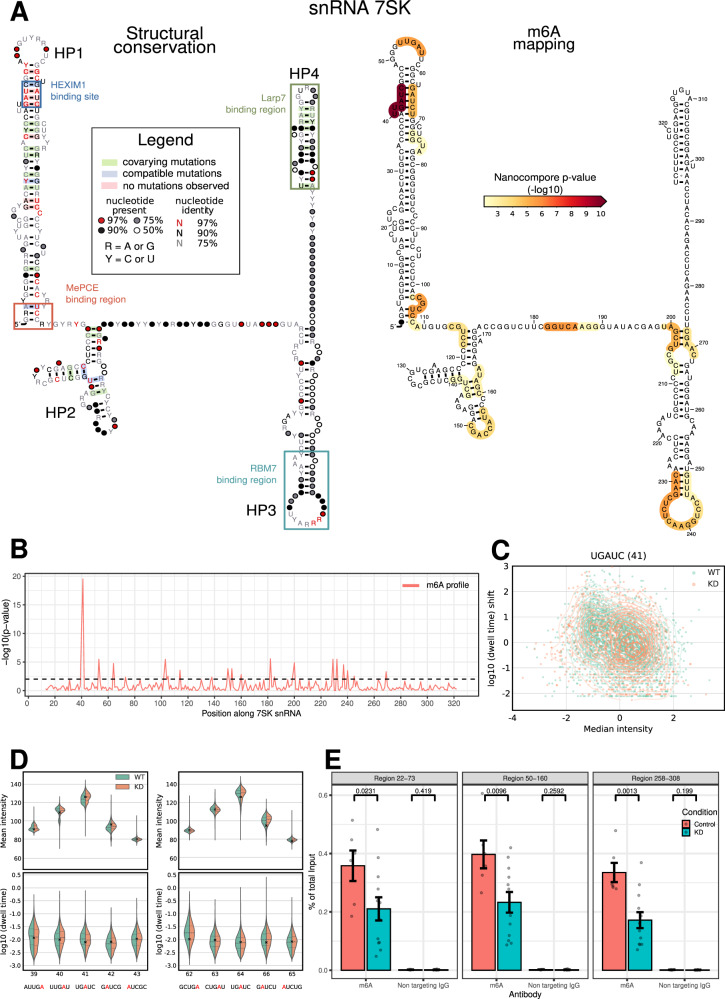
m6A identification in 7SK RNA. **A** On the left, the secondary structure of 7SK showing positions of known protein binding sites and structural conservation. On the right, the secondary structure of 7SK with the Nanocompore p-value (METTL3-KD vs WT, GMM-logit test) overlaid as a colour scale. For each nucleotide the colour indicates the lowest p-value among those of the 5 kmers that overlap it. Only *p*-values<0.01 are shown in colour. **B** m6A profile of 7SK, showing the Nanocompore GMM-logit p-value (y axis, -log10) across the transcript length. **C** Scatter plot showing the scaled median intensity vs the scaled log10 dwell time for each read covering kmer 41 of 7SK. Each point shows data for a distinct read colour coded according to the sample. The contour lines show the kernel density estimates for the two samples. For visualisation purposes the x- and y- axis are truncated at -4 and +3 respectively. **D** Violin plots showing the distributions of median intensity (top) and scaled log10 dwell time (bottom) for the Hexim1 binding sites and neighbouring kmers. All coordinates refer to the first nucleotide of each kmer relative to ENST00000636484. The cross mark indicates the intensity and dwell time value of the kmer according to the unmodified model. **E** m6A RIP-qPCR results in three non-overlapping regions of 7SK in WT and METTL3 KD MOLM13 cells. Bars show the mean of 6 independent experiments. Vertical bars show the standard error of the mean. The p-values were calculated using a one-sided Welch’s t-test. Full uncropped scans of Western Blots confirming METTL3 KD are shown in Figure S14.

Using the same inducible METTL3 KD and control cells as above, we next performed high-coverage targeted DRS of the human non-coding snRNA 7SK. To do so, we designed a custom nanopore sequencing adapter targeting the 3’ end of 7SK (see **Materials and Methods** and Supplementary Table 2). With this approach we achieved consistently high coverage in all the samples (average of 4,844 reads per sample). 7SK is a highly structured RNA with numerous binding sites for interacting proteins, which together form the 7SK snRNPs (Fig. 6A). Nanocompore analysis of 7SK in METTL3 KD cells identified 24 significant kmers across its entire sequence (*p*-value<0.01, Fig. 6A, B). The most significant hit falls in the UGAUC kmer at position 41 (Fig. 6A–D), which corresponds to the 5’ palindrome of the double-stranded and structurally conserved binding site for HEXIM1^36,37^. Interestingly, the 3’ GAUC palindrome at position 64 is also a significant site (Fig. 6A, B, D). These results suggest that the two central adenosines of the double stranded HEXIM1 binding site (A43 and A65) are both methylated by METTL3. We also identified 5 significant overlapping kmers between positions 229 and 250 in the terminal loop of hairpin 3 (HP3) (Fig. 6A, B). This region was recently shown to be the binding site for RNA-binding motif protein 7 (RBM7), which mediates the activation of P-TEFb by releasing it from 7SK snRNP, as well as for the structure- and context-specific binder hnRNP A1/A2^38,39^. We validated the presence of m6A in 7SK by RNA immunoprecipitation and qRT-PCR on the nuclear RNA fraction, finding a significant reduction of m6A enrichment upon METTL3 KD (Figs. 6E and  S14).

We next sought to extend our investigation of 7SK to include other modifications in addition to m6A. To this end we used IVT to generate large amounts of 7SK RNA devoid of all modifications. We then sequenced this IVT 7SK by DRS and analysed the resulting data with Nanocompore, using the dataset from targeted sequencing of WT MOLM13 cells as the reference condition. This approach potentially allows mapping of all RNA modifications in targeted RNAs, albeit without revealing the type of each modification. We identified 68 significant kmers spread across the entire 7SK sequence (1% FDR, Fig. S15A). The most significant region identified is ~10nt long and is located at the stem-loop boundary of HP3 (Fig. S15B). This region encompasses the m6A site identified at position A245 by the analysis of METTL3-KD, as well as a known Ψ site at position U250 (Fig. S15C)^40^. We also observed a significant change between IVT and WT RNA samples at A43 (UG**A**UC kmer, *p*-value=0.0608) and A65 (GCUG**A** and CUG**A**U kmers, *p*-values = 0.0839 and 0.0346, respectively), supporting the presence of the two m6A sites that we identified above in the double stranded HEXIM1 binding site.

## Discussion

In recent years substantial progress has been made in our understanding of the roles and functions of RNA PTMs. The diverse range of RNA PTMs biological roles are mediated by their capacity to dynamically regulate the physical and chemical properties of RNA molecules, for example by creating or masking binding sites, altering RNA structure or modulating expression and subcellular distribution^41,42^. However, fully understanding the breadth and scope of RNA modifications as well as their dynamic regulation in physiological and pathological contexts requires efficient and accurate methods to detect their presence and to map them to the respective RNA sequence contexts.

In this paper we introduce Nanocompore, a robust and versatile method for the identification of multiple types of RNA modification from Nanopore DRS data. Nanocompore performs a signal level comparison between two conditions, allowing identification of significant changes indicative of the presence/absence of RNA modifications (Fig. 1). Our approach has several advantages over alternative RNA PTM mapping methods. First, it is based on Nanopore DRS, a technique which is seeing rapid adoption and that, unlike previous genome-wide strategies, is not affected by reverse transcription or PCR amplification biases. Second, it maps RNA modifications in the context of long reads, giving critical information on RNA PTMs on individual gene isoforms. Third, our comparative strategy does not require any training and can be applied as-is to different RNA modifications, as long as a modification-depleted reference sample is available. Fourth, the approach implemented in Nanocompore is paving the way for future works to study RNA modifications at single molecule resolution. Finally, we implemented analysis pipelines in the Nextflow and Snakemake Domain Specific Languages, allowing automatic execution of all processing steps, from raw data up to the execution of Nanocompore and other RNA modification tools, thus greatly simplifying the bioinformatics work.

We extensively validated the performance of Nanocompore in silico, in vitro, and in vivo in both *ime4Δ* yeast cells as well as METTL3 KD human cells (Figs. 2–5). In both human and yeast, we were able to recapitulate previous observations on the distribution of m6A and provide new interesting insights. For example, we found m6A to be enriched toward mRNA stop codons as well as for the short motif DRACH. Furthermore, we confirmed with orthogonal techniques that m6A is enriched at the sites identified by Nanocompore both in human and in yeast. However—despite being greater than 20% in yeast—the overlap between Nanocompore and orthogonal techniques is incomplete, likely due to a combination of biological variability between samples as well as technical biases that affect the two technologies in different ways. In this regard, more work is still required in order to generate a reliable ground-truth annotation of m6A sites.

As an additional proof-of-concept, we performed high coverage targeted sequencing of non-polyadenylated ncRNAs, identifying multiple putative modification sites in the 7SK snRNA (Fig. 6). In addition to METTL3-dependent m6A sites we were also able to profile the overall modification landscape of 7SK by comparing our sample with an IVT control.

Through the creation of thousands of artificial datasets, we showed that Nanocompore performs well with mixed populations of modified/unmodified reads in the control and experimental samples. Although it is currently unsuitable for the identification of very low-frequency modifications, our benchmarks show that for abundant transcripts we achieve high sensitivity where as little as 20% of reads are modified. However, these simulations also show that the sensitivity is highly influenced by (a) expression level, (b) modification stoichiometry, and (c) efficiency of modification reduction in control. These observations strengthen the importance of having good control conditions (such as high efficiency knock-downs, knock-outs, or IVT samples) and high depth of sequencing. In our experiments, to profile m6A in yeast we achieved a median coverage of 120 reads per transcript. The True Positive Rate for m6A detection at this sequencing depth is ~48%, highlighting the fact that the low throughput of individual MinION flowcells currently does not provide enough coverage to resolve RNA modifications transcriptome wide. However, newer releases of DRS kits provide constant improvements in terms of throughput. Furthermore, when cost and amount of RNA are not limiting factors, users have the option of pooling multiple MinION flowcells or using a PromethION to achieve higher coverage. A further limitation that emerged from our benchmarks, which is intrinsic to the methods that directly use electrical signals to identify modifications, is the spatial resolution of modification calling. Since the reader head of the pore contains ~5 nucleotides, a single modified nucleotide can potentially affect the signal of up to 5 consecutive kmers, making it hard to resolve modification position with single nucleotide precision.

An additional feature of Nanocompore is that by analysing knock-down or knock-out samples it intrinsically assigns RNA modifications to specific writer enzymes, thus allowing to discern the individual roles of multiple enzymes that catalyse the same modification. It will also be of great interest to assess the effects of pharmacological inhibition of enzymes that regulate or deposit RNA modifications, for example in cancer, viral infections and potentially other diseases^43–45^. However, an important caveat to be considered when pursuing this approach—as well as any other method based on loss-of-function of catalytic enzymes—is that compensation between different enzymes or functional interactions between neighbouring modifications could be a confounding factors for Nanocompore analysis and currently cannot be accurately resolved solely with our method, in particular for long periods of inhibition of the RNA modifying enzymes. Because of this intrinsic inability of comparative methods to directly assign modifications, it is currently not possible to study multiple types of modifications at the same time.

An intrinsic feature of Nanocompore is its ability to assign modifications to specific isoforms, although this implies that Nanocompore requires either a well-annotated transcriptome or a custom transcriptome annotation generated from the DRS data. In addition, it is becoming increasingly important to obtain information about modification stoichiometry and combinatorics. Although Nanocompore currently does not allow measuring stoichiometry, one of its major advantages is the ability to detect RNA modifications at single molecule resolution. As a proof of concept we applied our analysis to the most significant m6A sites found by Nanocompore in β-actin mRNA and found that multiple methylated residues are present in the same molecule independently of one another at a given time. Although this type of analysis can not currently be applied transcriptome-wide, and although these results are still not quantitative in nature, they suggest the presence of highly site-selective intramolecular deposition and/or removal of m6A. This is the first observation of this kind to date, and it will need to be cross-validated when other methods enabling the same level of resolution become available.

The last few years have seen a remarkable increase in the number of methods available for modification detection from Nanopore data. The majority of these focus on the identification of only one type modification (typically m6A) whereas others, such as Nanocompore, NanoRMS, Epinano, and Eligos have been tested on a larger number of distinct modifications. The tools available also differ greatly in terms of methodology employed: for example, certain tools use machine learning algorithms (e.g. nanom6A, MINES, nanoDoc, Penguin, nano-ID, Epinano) whereas others apply clustering techniques and statistical testing (e.g. Tombo, Nanocompore, xPore, nanoRMS, Yanocomp, DiffErr, DRUMMER and Eligos). At the same time, these methods also differ in terms of strengths and shortcomings, which have been extensively reviewed in recent works^13^. Here we have benchmarked the performance of Nanocompore at detecting m6A against a small set of representative tools (namely Differr, Eligos2, Tombo, EpiNano and MINES), finding that in most situation Nanocompore achieves very high accuracy at the cost of lower sensitivity. Although this benchmark was done in yeast, we expect similar results for other species. However—as we and others^20^ have observed—low sequencing coverage negatively impacts modification detection. For this reason, a lower sensitivity can be expected for complex transcriptomes such as the human one. In addition, our experiments with synthetic RNAs also show that performance metrics are heavily influenced by modification stoichiometry and relative reduction of the modification in the control condition. Despite these observations, the field is still lacking a systematic comparison of the performance of all the methods available, of how it is impacted by the factors mentioned above and how it varies between different modifications or model species. For this reason, we recommend users seeking to detect RNA modifications from Nanopore data to test multiple methods that implement different approaches and to carefully assess the impact of coverage and knock-down/knock-out efficiency under their experimental settings.

In conclusion, Nanocompore offers a versatile, robust, and practical method to readily identify RNA modifications from Nanopore DRS experiments. Its adoption by the scientific community has already benefited a number of studies and should continue shedding light on the distribution and function of RNA modifications at high resolution, helping to reveal the currently hidden life of RNAs.

## Methods

### Cell culture and KD/KO experiments

The RNA from WT and METTL3 KD MOLM13 cells was obtained from Barbieri et al.^32^. Briefly, cells were cultured in RPMI1640 (Invitrogen) supplemented with 10% FBS and 1% penicillin/streptomycin/glutamine. Conditional knock-downs (KD) using METTL3-targeting or scrambled shRNAs were performed as previously described^32^. For lentivirus production, 293T cells were transfected with PLKO.1 lentiviral vector containing the shRNA sequences (Table S2), together with the packaging plasmids psPAX2 (Addgene Plasmid #12260), and VSV.G (Addgene Plasmid #14888) for METTL3 KD or Pax2 (Addgene Plasmid #35002), at a 1:1.5:0.5 ratio, using Lipofectamine 2000 reagent (Invitrogen) according to the manufacturer’s instructions. Supernatant was harvested 48 and 72 h after transfection. 1 × 10^6^ cells and viral supernatant were mixed in 2 ml culture medium supplemented with 8 μg/ml polybrene (Millipore), followed by spinfection (60 min, 900g, 32 °C) and further incubated overnight at 37 °C. The medium was refreshed on the following day and the transduced cells were cultured further. MOLM13 cells (5 × 10^5^) were infected using PLKO-TETon-Puro lentiviral vectors expressing shRNAs. After 24 h of infection, the cells were replated in fresh medium containing 1 μg/ml of puromycin and kept in selection medium for 7 days. shRNA expression was induced by treatment with 200 ng/ml doxycycline for 4 days for METTL3 KD. Near complete loss of METTL3 RNA and protein was confirmed by Western Blot and qPCR by Barbieri et al.^32^. For METTL3 knock-out (KO) experiments, lentiviruses were produced in HEK293 cells using ViraPower Lentiviral Expression System (Invitrogen) according to manufacturer’s instructions. MOLM13 cells stably expressing Cas9 were transduced with lentiviral gRNA vectors expressing either empty or METTL3 gRNAs (Table S2) and selected with puromycin from day 2 to day 5. At day 5 post-transduction, the cells were suspended in fresh medium without puromycin. At day 6, cells were harvested for RNA extraction.

The diploid *S. cerevisiae* strains used for generating the *ime4Δ* mutant were derived from the SK1 background. The *ime4Δ* strain was generated using the one-step gene replacement method described previously^46^.

### RNA purification and  in vitro transcription

Total RNA was isolated from MOLM13 cells using the RNeasy midi kit (Quiagen) and polyA+ RNA was purified from 30 μg total RNA using the Dynabeads mRNA Purification Kit (Thermo Fisher Scientific) according to the manufacturer’s instructions. For production of unmodified 7SK RNA, synthetic double stranded DNA template for in vitro transcription (IVT) was produced by hybridization of synthetic Megamer® Single-Stranded DNA Fragments (IDT) containing the 7SK sequence downstream of a T7 promoter (Table S3). 500ng of double stranded DNA template were used in 20 μl IVT reactions for 1h using the TranscriptAid T7 High Yield Transcription Kit (Thermo Fisher Scientific), following the manufacturer’s instructions. The RNA product was purified using the RNA Clean & Concentrator kit (Zymo Research). Wild Type and *ime4Δ* yeast cells were collected after 4h in sporulation medium and total RNA was extracted with acid phenol:chloroform:isoamyl alcohol as previously described^47^. polyA+ RNA was purified from total RNA using the Dynabeads mRNA Purification Kit (Thermo Fisher Scientific) as above.

### miCLIP

miCLIP was performed in duplicates with RNA isolated from wild type and METTL13 KO MOLM13 cells. The protocol is conceptually related to the original m6A miCLIP protocol^48^, but uses total RNA as input and follows a more recent variant of iCLIP protocol^49^. 4 μg of total RNA were fragmented with RNA fragmentation reagents (ThermoFisher) following the manufacturer’s instructions. Fragmented RNA was then incubated with 2.5 μg anti-m6A antibody (Abcam, ab151230) in IP buffer (50 mM Tris-HCl pH 7.4, 100 mM NaCl, 0.05% NP-40) at 4 °C for 2 h, in rotation. Subsequently, the solution was placed in 6-well plates on ice and irradiated twice with 0.3 J cm−2 UV light (254 nm) in a Stratalinker crosslinker. 30 μl protein G beads (Dynabeads) per sample were washed twice with IP buffer and then incubated with the RNA-antibody solution at 4 °C for 1.5 h, in rotation. After the IP, the RNA-antibody-beads complexes were washed twice with High-Salt Wash buffer (50 mM Tris-HCl pH 7.4, 1M NaCl, 1 mM EDTA, 1% Igepal CA-630, 0.1% SDS, 0.5% sodium deoxycholate), once with IP buffer and once with PNK Wash buffer (20 mM Tris-HCl pH 7.4, 10 mM MgCl_2_, 0.2% Tween-20). The beads then proceeded to 3′ dephosphorylation and the rest of the iCLIP protocol. The 3’ adapters for on-bead ligation carry the sequences found in Table S4. Samples were mixed after the adapter removal step. Following the SDS-PAGE gel, the membrane was cut from 45 kDa to 185 kDa and RNA was extracted. The following sequence of the RT primer was used: /5Phos/WWW CGTAT NNNN AGATCGGAAGAGCGTCGTGAT/iSp18/GGATCC/iSp18/TACTGAACCGC. cDNA libraries were sequenced with single end 100bp reads on Illumina HiSeq4000.

### Nanopore direct-RNA sequencing (DRS)

RNA sequencing was performed following the instruction provided by Oxford Nanopore Technologies (Oxford, UK), using R9.4 chemistry flowcells (FLO-MIN106) and direct-RNA chemistry sequencing kits (SQK-RNA001 or SQK-RNA002). For polyA+ transcriptome sequencing, we followed the conventional DRS protocol using the provided polyT (RTA) adapter. For the targeted sequencing, we ordered custom reverse transcription adapters complementary to the 3’ end of 4 selected noncoding RNAs, and followed the sequence-specific DRS protocol (Table S5). For library preparation of IVT 7SK, we used 500ng of unmodified IVT RNA prepared as described above, using the adapter complementary to the 3′end of 7SK.

### m^6^A RNA immunoprecipitation and qRT-PCR

Cell nuclei were obtained from MOLM13 WT (six independent biological replicates) or METTL3-KD cells (six independent biological replicates for each shRNA) six days after doxycycline administration. Cell lysis was performed in 10 mM TRIS pH = 7.8, 140 mM NaCl, 1.5 mM MgCl_2_, 10 mM EDTA, 0.5% NP40 and RNase inhibitor (RNaseOUT™, Thermo Fisher Scientific, 10777019, lot # 2232786) for 30 min on ice followed by centrifugation at 3,000 × *g* for 3 min. Nuclear RNA fraction was then purified using the RNAeasy midi kit (Qiagen). Successively, 4 μg of nuclear RNA were fragmented for 3 min and 30 second at 70 °C using the RNA fragmentation Reagents (Thermo Fisher Scientific, AM8740, lot # 00786992). Fragmented nuclear RNA was then purified using the RNA Clean & Concentrator™-5 kit (Zymo Research, R1016). meRIP qRT-PCR was performed, as previously described^50^ with some modifications. Briefly, for each immunoprecipitation reaction 1 μg of fragmented nuclear RNA was incubated 2 h at 4 °C in rotation with anti-m^6^A (Abcam, ab151230, lot #GR3319501-1) or anti-GFP Antibodies (Abcam, ab290, lot #GR3321575-1) in a final volume of 1ml RIP Buffer (RIP buffer 5×, ddH_2_O, RNaseOUT™)^50^, and subsequently incubated 2h at 4 °C in rotation with 50 µL of BSA-coated Dynabeads G (Thermo Fisher Scientific, 10004D). A total of 5% of each immunoprecipitation reaction was saved as input control. To elute RIP-RNA, beads were incubated twice 30 min at 37 °C in a thermo-shaker (1100rpm) in 40 μl of elution buffer (RIP buffer 1×, 6.7 mM N6-Methyladenosine 5′-monophosphate (Santa Cruz Biotechnology, sc-215524, lot # L1820), RNaseOUT™). Input and RIP samples were finally purified using the RNA Clean & Concentrator™-5 kit (Zymo Research, R1016). cDNA was obtained using the high-capacity cDNA reverse transcription kit (Thermo Fisher Scientific, 4368814). The levels of 7SK were measured using a QuantStudio 6 Flex real-time PCR machine and PowerUp™ SYBR™ Green PCR master mix (Thermo Fisher Scientific, A25780) according to the manufacturer’s instructions. Statistical testing for differences between KD and Control was done with the one-tailed Welch’s t-test. qRT-PCR primers: 7sk (22-73): Fwd 5′-GCGACATCTGTCACCCCATT-3′; Rev 5′-CAGCCAGATCAGCCGAATCA-3′. 7sk (50-160): Fwd 5′-GGGTTGATTCGGCTGATCT-3′; Rev 5′-GGGGATGGTCGTCCTCTT-3′ 7sk (258-308): Fwd 5’-CGTAGGGTAGTCAAGCTTCCA-3’; Rev 5’-CAGCGCCTCATTTGGATGTG-3′

### Western blotting

Western blot experiments were performed as previously described (Barbieri, Nature 2017) using the following antibodies: anti-METTL3 (Abcam, ab195352, lot #GR3247121-3) and anti-beta Actin (Abcam, ab8227, lot #GR3255609-1).

### In silico simulated datasets

#### Unmodified RNA model

We used an in vitro transcribed human RNA DRS dataset released by the Nanopore WGS consortium as a ground truth for non-modified RNA bases (https://github.com/nanopore-wgs-consortium/NA12878). This dataset contains all possible 5-mers on average 58,307 times. The reads were aligned on gencode release 28 human reference transcriptome with Minimap2 v2.14 and we realigned the signal to the reference sequence using *Nanopolish eventalign v0.10.1* followed by *NanopolishComp Eventalign_collapse v0.5* . Next, we collected the median intensity and dwell time data for each 5mers and tried to fit 44 different distributions. We selected distributions minimising the sum of square root error for all kmers between the observed and modelled data. In addition, we also based our selection on the possibility to easily change the parameters of the distributions to simulate the presence of modifications. We selected the Wald distribution and the Logistic distribution for dwell time and median intensity, respectively. Finally, we generated a model file containing the parameters of the observed and model distributions for each 5-mer. The up-to-date model file is distributed with Nanocompore. The detailed analysis is available in the following Jupyter notebook: https://github.com/tleonardi/nanocompore_paper_analyses/blob/master/in_silico_dataset/01_IVT_Kmer_Model.ipynb.

#### Simulated reference sequence

We generated a set of in silico reference sequences. In order to maximise the sequence diversity and kmer coverage we used a “guided” random sequence generator. In brief, the sequences are generated base per base using a random function, but the program keeps track of the number of times each kmer was already used. The sequence is extended, based on a random function with a weighted probability for each kmer inversely proportional to their occurrence in the sequences already generated. This ensures that all kmers are represented as uniformly as possible, but it leaves some space to randomness. We generated a set of 2000 sequences 500 bases long each maximising the 9-mers coverage. We excluded any homopolymers longer than 5 bases, as they are likely to be miscalled in nanopore data. Kmer coverage in the final sequence set are summarised in Table S6. The detailed analysis is available in the following Jupyter notebook: https://github.com/tleonardi/nanocompore_paper_analyses/blob/master/in_silico_dataset/02_Random_guided_ref_gen.ipynb.

#### Simulated modified and unmodified datasets

*Nanocompore* comes with a companion tool called *SimReads* which can generate simulated read data based on a fasta reference and a kmer model file. Essentially, *SimReads* walks along the reference sequence and generates intensity and dwell time values corresponding to each 5-mers. To do so, it uses a probability density random generator using the kmer model values (location and scale) bounded by the extreme observed values. This tool can also offset the model mean by a fraction of the distribution standard deviation to simulate the effect of RNA modifications. This can be done for all the reads or only on a subpopulation of reads. *SimReads* generates files similar to the output of *NanopolishComp EventalignCollapse*. This means that the datasets can be directly used as input for *NanoCompore SampComp*. Using Nanocompore v1.0.0rc3 with the previously described simulated reference sequence set we generated 144 in silico datasets with various amplitude of modification of the median signal intensity and the dwell time (0, 1, 2, 3, and 4 standard deviation) as well as different fractions of modified reads (10%, 25%, 50%, 75%, 90%, and 100%). All the datasets were simulated in duplicate with a uniform coverage depth of 100 reads. The detailed analysis is available in the following Jupyter notebook: https://github.com/tleonardi/nanocompore_paper_analyses/blob/master/in_silico_dataset/03_Simulated_dataset_gen.ipynb.

#### Analysis of simulated datasets

We compare the 144 datasets containing simulated modifications against the reference dataset generated from the unmodified model with Nanocompore v1.0.0rc3 (See Nanocompore section after). The analysis was performed with all the statistical methods supported by *Nanocompore* using a sequence context of 2 nucleotides (https://github.com/tleonardi/nanocompore_paper_analyses/blob/master/in_silico_dataset/04_nanocompore.sh). The result database was subsequently parsed and the predicted modified sites were compared with the position of the known simulated positions. A hit was considered true positive (TP) when we found a significant p-value within 3 nucleotides of a known modified position. A significant hit outside of this window was counted as a false positive (FP). Finally, we plotted the Receiver Operating Characteristic (ROC) curves corresponding to the TP rate compared with the FP rate for every Nanocompore comparison performed (https://github.com/tleonardi/nanocompore_paper_analyses/blob/master/in_silico_dataset/05_calc_roc.sh).

#### Sequencing and analysis of synthetic modified oligos

The four PAGE-purified, synthetic oligonucleotides of 100nt were ordered through Horizon Discovery LTD at a concentration of 0.2 µmol. Oligo1, 2, and 3 carried 3 modified nucleotides each, whereas Oligo4 was the unmodified control. All the oligonucleotides have the same sequence, but they contain different modifications sufficiently spaced (23 bases) to avoid interactions between modifications. The sequence was chosen in order to combine all the know consensus of the modifications in a single oligo sequence in order to be able to use a single non-modified reference for all oligos:

- m6A: GGACU (strong DRACH consensus)
- m6A: CGACC (Weak NRACH consensus)
- m6A: CUAGC (Anti DRACH consensus)
- Inosine: UUAGC (loose motif in editing-enriched regions (EERs) – from Blango and Bass 2016, and Eggington et al. 2011).
- PseudoU: UGUAG (from Pus7’s UGΨAR motif, and 7SK IVT peak…)
- m62A: GUGAACC (from the 18S rRNA modified sequence)
- m5C: CCCGGG (from Huang et al. 2019)
- m1G: CAGGTCG (from the tRNA m1G37 position)
- 2’OmeA: GAGAGAA (from rRNA doi: 10.1093/nar/gkw810)

The motifs were all expanded to 7 bases and combined in a sequence separated by a randomly generated buffer of 9 bases. We generated all possible permutations of the blocks and 1000 different versions of the randomly generated buffer sequences (disallowing homopolymers), totalling 216,000 candidate sequences. We then computationally folded all of the candidate sequences using RNAfold v2.4.15 from the Vienna package. Finally, we calculated a combined score taking into account the folding score and the base composition balance and picked the best candidate:

m6A_strong-Inosine-m62A-m6A_anti-m5C-m1G-m6A_weak-PseudoU-2OmeA|seed=802

>control

AUACUCGACAUAGAUAGGACUCUUUAGCUAGUGAACCCUAGCCUCCGGAGACAGGUCGCGACCUGUGUAGAUGAGAGAACUGAGUGCACAAAAAAAAAAA

>mod_set_1

AUACUCGACAUAGAUAGG(m6A)CUCUUUAGCUAGUGAACCCU(m6A)GCCUCCGGAGACAGGUCGCG(m6A)CCUGUGUAGAUGAGAGAACUGAGUGCACAAAAAAAAAAA

>mod_set_2

AUACUCGACAUAGAUAGGACUCUUU(I)GCUAGUGAACCCUAGCCUC(m5C)GGAGACAGGUCGCGACCUGUG(PseudoU)AGAUGAGAGAACUGAGUGCACAAAAAAAAAAA

>mod_set_3

AUACUCGACAUAGAUAGGACUCUUUAGCUAGUG(m62A)ACCCUAGCCUCCGGAGACAG(m1G)UCGCGACCUGUGUAGAUGAG(2’OmeA)GAACUGAGUGCACAAAAAAAAAAA

The full design analysis is now provided in the online companion analysis repository https://github.com/tleonardi/nanocompore_paper_analyses/tree/master/control_oligos_design

DRS libraries were prepared from 500 ng of each oligo using the SQK-RNA002 kit (ONT) and following the standard protocol. Libraries were then sequenced in individual FLO-MIN106 flowcells on a GridION instrument. The data was then basecalled with Guppy (v3.2.10) with default parameters. A known limitation of DRS is the poor data normalisation for short reads. To overcome this limitation and reduce noise, we only retained for further analysis the Guppy pass reads of at least 100nt in length (i.e., full length oligos and fusion reads). Filtered reads were then mapped to the reference unmodified sequence using minimap2 (-k 9 -m 5), the signal data was then resquiggled with Nanopolish and the aligned events table was collapsed with NanopolishComp as outlined before. The filtered datasets for Oligo1, 2, and 3 were then analysed with Nanocompore (v1.0.0rc3, -min_coverage 30, -downsample_high_coverage 5000). The Nanocompore signal peaks were generated as described in Peak Calling section using a p-value threshold of 0.01.

We then generated artificial datasets containing variable fractions of unmodified and modified reads, covering all possible combinations of 3 factors:

1. **f**, the fraction of modified reads in experimental condition, ranging from 0 to 1 in 0.1 increments
2. **r**, the fraction of modification reduction in control condition. Values 1, 0.8 or 0.5
3. **n**, the read coverage ranging from 16 to 4096 and doubling at each step.

For each dataset to be generated, we created 4 NanopolishComp index files:

1. A file referencing a random sample of n*f reads from the dataset containing the modification
2. A file referencing a random sample of n*(1-f) reads from the unmodified dataset
3. A file referencing a random sample of n*f*r reads from the dataset containing the modification
4. A file referencing a random sample of n*(1-f*r) reads from the unmodified dataset

This procedure was repeated 100 times for each combination of n, f, and r and analysed in 81.000 distinct Nanocompore runs using the combined files 1 and 2 as the experimental sample and the combined files 3 and 4 as the reference sample. We then analysed the results of Nanocompore in order to calculate, for each combination of n, f, and r, the mean number of True Positives, False Positives, True Negatives, and False Negatives identified. For this purpose, True positives were defined as the number of known modification sites with at least 1 significant kmer; False positives were defined as the number of significant kmers outside of the known modification sites; True negatives as the number of known unmodified sites that didn’t have any significant kmer and False negatives as the number of known modification sites not supported by any significant kmer.

### Direct-RNA datasets analysis

#### Reference files

For this study we used the following Human reference files all obtained from Ensembl:

- Human Genome reference: Human genome assembly GRCh38.p12
- Human Annotation reference: Ensembl Gene build release-97
- Yeast SK1 Genome Reference: http://cbio.mskcc.org/public/SK1_MvO/

### Data preprocessing

All the datasets were preprocessed using an automated analysis NextFlow pipeline, before running Nanocompore (https://github.com/tleonardi/nanocompore_pipeline). Raw reads FAST5 files were basecalled with ONT Guppy v3.1.5 and the basecalled reads were saved in FASTQ format. A post-basecalling quality control was performed with pycoQC (v2.2.4)^51^ to verify the consistency of the sequencing runs. A transcriptome reference FASTA file was created from the annotation BED file and genome FASTA file with Bedparse (v0.2.2)^52^. Reads were then aligned on the transcriptome reference with Minimap2 (v2.16)^53^ in unspliced mode (-x map-ont). The resulting aligned reads were filtered with samtools (v1.9)^54^ to keep only primary alignments mapped on the forward strand (-F 2324) and the raw signal was realigned on reads using Nanopolish eventalign (v0.11.1)^55^. Finally, the data was processed by NanopolishComp Eventalign_collapse (v0.6.2)^56^ to generate a random access indexed tabulated file containing realigned median intensity and dwell time values for each kmer of each read.

### Signal comparison with nanocompore

Nanocompore is a Python3 package dedicated to comparative analysis of DRS nanopore sequencing raw signal in order to identify potential RNA modification sites. Signal analysis and complex statistical tests are generally resource-intensive, but Nanocompore takes advantage of a multiprocessing architecture to process transcripts in parallel and has a relatively small memory footprint. Nanocompore requires at least 1 indexed tabulated file generated with NanopolishComp Eventalign_collapse for each of the 2 conditions to compare. The program will run with a single replicate per condition, but we recommend at least 2 to take full advantage of the advanced statistical framework. The analysis flow is divided in three steps: (1) white-listing of transcripts with sufficient coverage, (2) parallel processing and statistical testing of transcripts position per position, (3) post-processing and saving.

#### Transcripts whitelisting

In order to reduce the computational burden, Nanocompore first filters out transcripts with insufficient coverage. This is achieved by a rapid tally of reads mapped per transcripts followed by selection of transcripts having at least 30 reads mapped in all of the samples provided. Users can modify the threshold but the default value allows to get reproducible results. Optionally, one can provide a custom list of transcripts to include or exclude.

#### Statistical analysis

White-listed transcripts are processed in parallel to take advantage of multi-threaded architecture. First, the data corresponding to the reads mapped on each transcript is loaded in memory and transposed in the transcript space in a position-wise fashion. The current implementation of Nanocompore only uses the median signal intensity and the scaled log10 transformed dwell time, but the framework is flexible enough to aggregate more variables, such as the error rate or additional Nanopolish HMM states. The 2 experimental conditions are compared positions per position using a range of statistical tests. We included the Kolmogorov-Smirnov (KS) test as a robust univariate pairwise statistical test on current intensity and dwell time. These tests are performed independently on the median intensity and the dwell time. We also implemented a Gaussian mixture model (GMM) clustering-based method. For a given position we fit a bivariate 2 components GMM to all the data points observed (x=median intensity, y=dwell time), irrespective of the sample label. We then assign each data point to one of the two clusters and test for differences in the distribution of reads between clusters across conditions. To this purpose, testing is implemented in two ways: 1) by default, we fit a Logit model to the data using the formula *predicted_cluster~1+sample_label* and report the coefficient’s p-value. 2) As an optional alternative we do a one-way ANOVA test comparing the log odds of data points belonging to cluster one between the two conditions. After testing, it’s optionally also possible to aggregate the p-values of neighbouring kmers to account for the fact that modified bases affect the signal of multiple kmers. To this end, and due to the fact that neighbouring p-values are non-independent, we implemented in python a method that extends the Fisher’s statistic X=-2log(P_1_^w1^ P_2_^w2^ … P_k_^wk^) to approximate the distribution of the weighted combination of non-independent probabilities^26^. The combined p-values are computed all along the sequence using a sliding window of a given length. This method greatly reduces the prediction noise (false positive rate) at the expense of spatial resolution, while giving more weight to sites for which the effect of RNA modifications on the signal is spread over several kmers.

#### Post-processing, saving and data exploration with Nanocompore interactive plotting API

Results generated by the statistical module are collected and written in a simple key/value GDBM database. Although this data structure has limitations in terms of portability and concurrent access, it is natively supported by python and allows storing complicated data structures. For each test previously performed p-values are temporarily loaded in memory and corrected for multiple tests with the Benjamini-Hochberg procedure. Users can then obtain a tabulated text dump of the database containing all the statistical results for all the positions in the transcripts space or a BED file with the positions of significant hits found by Nanocompore converted in the genome space. Finally, we provide a convenient python wrapper over the GDBM database, allowing users to interactively access simple high level functions to plot and export the results (https://nanocompore.rna.rocks/demo/SampCompDB_usage/). The wrapper was initially developed for Jupyter but can essentially work with any python IDE. At the time of publication the wrapper allows to generate 6 different types of publication ready plots for a given transcript including (1) the distribution of p-values, (2) the distribution of signal intensity and dwell time, (3) the overall coverage per sample, (4) the nanopolish HMM states, (5) the kernel density of the signal and dwell time for a specific position and (6) the sharkfin plot of the p-values compared with Log Odds Ratio (for the GMM method).

### Downstream analyses

The code for all generic analyses, plots and metrics is available at https://github.com/tleonardi/nanocompore_paper_analyses/. The transcript intersection plot for the MOLM13 polyA dataset had been generated with UpsetR^57,58^.

#### Peak calling

Given that a single modification can affect the signal for multiple overlapping kmers, we developed a peak calling method to refine our predictions. Briefly, we first converted p-values in -log10 so that peaks correspond to positions with higher probability of being modified. We then defined a dynamic threshold per transcripts corresponding to the median of all the values above 2 (p-values <0.01). In the case where no significant p-values were found, the threshold was set to 2. Peaks were called using *scipy.signal*.find_peaks using the dynamic threshold described before as a minimal height and a minimal distance of 9 between 2 peaks (5 overlapping 5-mers). Examples can be found in Figure S12.

#### Metagene m6A coverage

The metagene m6A coverage analysis was done considering all nanocompore kmers with GMM logit p-value<0.01 and a log odds ratio >0.5. The plot was produced in R/Bioconductor with the Guitar package using the TxDb.Hsapiens.UCSC.hg38.knownGene package for the human transcriptome annotation and the SK1 reference transcriptome GFF for the yeast annotation.

#### Motif enrichment analysis of m6A sites

For the motif enrichment analysis of m6A sites identified by Nanocompore analysis of METTL3 KD, we extracted the sequence of all kmers tested by Nanocompore and having a p-value<0.5 (GMM-logit). The sequences were then sorted by p-value and analysed with Sylamer for the identification of over-represented words, using a word size of 5 and a growth parameter of 100. The Sylamer results were then imported in R for plotting. To produce a combined profile of the DRACH motif, the per-window p-values of all DRACH kmers were combined using Fisher’s method. For visualisation purposes, the final plot only reports the lines for the top 100 motifs with the greatest area under the sylamer curve, with the top one represented in colour.

#### Single molecule identification of m6A sites

To assign an m6A probability at A652, A1324 and A1535 for each read covering the β-actin transcript, we developed a dedicated post-processing script available at https://github.com/tleonardi/nanocompore_paper_analyses/m6acode/parse_sampcomdb.py. Briefly, for each of the three positions of interest, we extract the GMM model saved in sampCompDB, and for each read we then predict the probability that it belongs to each of the two clusters. To define which of the two clusters corresponds to m6A modified reads, we consider which of the two clusters has negative log odds of data points belonging to it in the KD condition (i.e., we consider which of the two clusters shrinks in the KD condition). To test the independence of the methylation events at these three sites, we performed a chi-squared test of independence comparing the expected number of molecules for each of the 8 combinations of modifications to the observed number of molecules. The results reported are obtained using a probability threshold of 0.75 (as predicted by the GMM) to consider a read as methylated. However, to ensure robustness of these results, the chi-squared test was repeated for all thresholds between 0.1 and 1 (0.05 steps) and p-values were adjusted accordingly using the Benjamini–Hochberg procedure. Adjusted p-values were >0.39 for all thresholds used.

#### 7SK structures

The 7SK multiple alignments and consensus secondary structure were obtained from Rfam (RF00100). Secondary structure plots were produced with R2R^49,59^ and a custom python script to annotate p-values as colour shading (available at https://github.com/tleonardi/nanocompore_paper_analyses/blob/master/ncRNAs_structures/create_annotations.py)

### miCLIP analysis

miCLIP data and corresponding input data was analysed using the iMaps web server (https://imaps.genialis.com/). Briefly, raw reads were demultiplexed and trimmed (for adaptors and quality), before being mapped to a tRNA and rRNA index using STAR (v2.4.0.1)^60^. Unmapped reads were then mapped to GRCh38 GENCODE primary assembly, using GENCODE annotation v30. STAR parameter -alignEndsType Extend5pOfRead1 was used to ensure no soft clipping of cDNA start sites. PCR duplicates were removed based on unique molecular identifier (UMI) and mapping position. cDNA start -1 positions were taken as crosslink sites. Significant Nanocompore clusters were determined by merging overlapping kmers with a GMM_logit_contex_2 p-value < 0.001 using bedtools merge (v2.28.0). Control sites were selected as those with a context 2 p-value of 1 and split into those that did or did not contain DRACH within a 100nt window around the center of the cluster. Due to large differences in library size, miCLIP crosslinks were first filtered to remove intergenic and ncRNA sites and then subsampled using GNU coreutils shuf, to generate libraries equal in size to the smallest library, totalling 47,012 crosslinks. Crosslink counts were divided by gene TPMs calculated from either WT or KO mock miCLIP samples. BigWig files were generated from the normalised bedgraphs, which were used as the input to deepTools^61^ (v3.3.0) computeMatrix and plotHeatmap to generate metaprofiles -1000 to +1000bp around the center of Nanocompore clusters with a bin size of 2bp. The resulting tabular output was further analysed in R. Shaded regions on the plot represent the mean +/- the standard deviation at each position in the profile (WT miCLIP n=4, KO n=2). Both the mean and bounds were smoothed using loess regression with a span of 0.6. In order to test for a significant difference between WT and KO profile, mean values from WT and KO miCLIP between positions -20 to +20 around nanopore sites were subjected to a Mann–Whitney U test.

### Modification prediction Comparison with MetaCompore

In order to compare Nanocompore against most of the other tools available for RNA modification detection in a reproducible way, we wrote a snakemake pipeline called MetaCompore (https://github.com/a-slide/MetaCompore). For this study, we used MetaCompore v0.1.2, which includes the latest version of following tools : Epinano 1.2.0, Eligos 2.0.0, Tombo 1.5.1, differr_nanopore_drs (latest version), Mines (latest version) and Nanocompore 1.0.3. MetaCompore preprocess the data for all the tools, including Basecalling with ONT-Guppy 4.2.2 (except for Epinano which required the older 3.1.5 version), read alignment to the reference transcriptome with Minimap2 2.17, alignments filtering with pyBiotools 0.2.7 and signal realignment with f5c 0.6. For portability and reproducibility reasons, every module of MetaCompore is provided within its own singularity container and all the options used for a run are tracked in a YAML configuration file. Nanocompore and Epinano are the only tools to support experimental replicates. For all the other tools we merged the data obtained from replicates. Since every tool outputs a different kind of statistics/format, MetaCompore filters the data following the respective authors recommendations and when possible converts the result in a similar format containing the significant site associated with their p-value and Effect size. For Nanocompore and Tombo which both work in signal space, we added a peak calling denoising step to narrow down the results.

For the comparison in this paper we used a Yeast SK1 dataset comparing 2 replicates of WT yeast against 2 replicates of an IME4 KO mutant (m6A writer in Yeast). We used the Yeast SK1 reference transcriptome (https://www.yeastgenome.org/strain/SK1). Prior to modification detection, we ran an optional pipeline step to filter out any reference transcript with less than 30 reads in all replicates. The command line options used for all the tools are available in the MetaCompore configuration file provided as supplementary material.

### Benchmarks of MetaCompore results against known yeast m6A sites

We compiled an orthogonal reference set of m6A sites from SK1 yeast by taking m6A-Seq sites from Schwartz et al.^30^ and MAZTER-seq sites from Garcia-Campos et al.^29^. The sites and surrounding sequence were mapped to the MvO SK1 genome fasta to obtain the equivalent genomic coordinates. ACA sites annotated with MAZTER-seq confidence group > 1 or supported by m6A-Seq were taken as single nucleotide positions. Non-ACA sites were taken from m6A-Seq. If an m6A-Seq window overlapped with one or more single nucleotide sites it was removed from the reference set. In total this produced a set of 882 single nucleotide positions, and 415 80nt windows, amounting to 1297 reference m6A positions.

The comparison of each method in MetaCompore with this orthogonal reference dataset was based on our *S. cerevisiae* DRS data and limited to transcripts with a coverage of at least 30 reads. The calculations of the TPR/FPR/F1 score/Precision of each method was done at a p-value threshold of 0.01. For each method we constructed a confusion matrix using the following criteria:

- True Positives: the number of ground-truth m6A sites overlapping at least one significant kmer according to the given method. The True Positive Rate was further defined as the number of True Positives divided by the total number of m6A sites in the ground-truth set.
- True Negatives: the number of not significant DRACH kmers in the transcriptome (limited to transcripts present in the DRS dataset)
- False Positives: the number of significant kmers that do not overlap a ground-truth m6A site. The False Positive Rate was further defined as the number of False Positives divided by the sum of False Positives and True Negatives.
- False Negatives: the number of ground-truth m6A sites not overlapped by any significant kmer

For the purposes of the calculations above, we used the results tables produced by each method (prior to Metacompore postfiltering) and applied the following criteria to consider a kmer as significant:

Eligos2: reported p-value<=0.01 and odds ratio>1.2 (as recommended by the authors)

Diff_err: reported *p*-value<0.01 (diff_err results are already filtered by *p*-value and G-test)

MINES: all sites (MINES only reports significant sites)

Epinano: sites classified as modified (modification probability >0.5)

Tombo: reported *p*-value<0.01 after Benjamini-Hochberg adjustment

Nanocompore: reported *p*-value<0.01 and GMM log odds ratio>0.5 (for GMM method only)

For the benchmarks above, the single nucleotide sites identified by each method were extended to 10nt prior to overlapping them with the ground-truth set.

### Reporting summary

Further information on research design is available in the Nature Research Reporting Summary linked to this article.

## Supplementary information

Supplementary Information

Reporting Summary

## Data Availability

The direct RNA and miCLIP datasets data generated in this study have been deposited in the European Nucleotide Archive database under accession codes PRJEB44511 and PRJEB35148. The data supporting the findings of this study are available from the corresponding authors upon reasonable request.

## Code availability

The computational methods and custom scripts used for this paper are available in the following Github repository: https://github.com/tleonardi/nanocompore_paper_analyses. The code of the Metacompore pipeline is available in the following Github repository: https://github.com/a-slide/MetaCompore
