# Supplementary material for: RNA modifications detection by comparative Nanopore direct RNA sequencing
Source: Nat Commun. 2021 Dec 10;12:7198. doi: 10.1038/s41467-021-27393-3 (PMC8664944; doi:10.1038/s41467-021-27393-3)
Supplement: Supplementary file 1 — Supplementary Information [file 41467_2021_27393_MOESM1_ESM.pdf]

RNA modifications detection by comparative Nanopore direct RNA sequencing

Adrien Leger[1*], Paulo P. Amaral[2,3,4*], Luca Pandolfini[2,5], Charlotte Capitanchik[6], Federica Capraro[6,7], Valentina Miano[10], Valentina Migliori[2], Patrick Toolan-Kerr[6,7], Theodora Sideri[6], Anton J Enright[11], Konstantinos Tzelepis[2], Folkert J. van Werven[6], Nicholas M. Luscombe[6,8,9], Isaia Barbieri[2,10], Jernej Ule[6,7], Tomas Fitzgerald[1], Ewan Birney[1**], Tommaso Leonardi[2,12**] and Tony Kouzarides[2**]

[1] *European Molecular Biology Laboratory, European Bioinformatics Institute, Wellcome Genome Campus, Hinxton, Cambridge, UK*
[2] *The Gurdon Institute, University of Cambridge, Tennis Court Road, Cambridge, CB2 1QN, UK*
[3] *The Milner Therapeutics Institute, Jeffrey Cheah Biomedical Centre, University of Cambridge, Puddicombe Way, Cambridge, UK*
[4] *INSPER - Institute of Education and Research, Rua Quatá 300, São Paulo, SP, 04546-042, Brazil*
[5] *Istituto Italiano di Tecnologia (IIT), Center for Human Technologies (CHT), 16152 Genova, Italy*
[6] *The Francis Crick Institute, London NW1 1AT, UK*
[7] *Department of Neuromuscular Diseases, UCL Queen Square Institute of Neurology, Queen Square, London, WC1N 3BG, UK*
[8] *Department of Genetics, Environment and Evolution, UCL Genetics Institute, London, UK*
[9] *Okinawa Institute of Science & Technology Graduate University, Okinawa, Japan*
[10] *Department of Pathology, Division of cellular and molecular pathology, lab block level 3, box 231, Addenbrooke's hospital, CB2 0QQ, Cambridge, UK*
[11] *Department of Pathology, University of Cambridge, Tennis Court Road, Cambridge, CB2 1QP, UK*
[12] *Center for Genomic Science of IIT@SEMM, Istituto Italiano di Tecnologia (IIT), 20139 Milan, Italy*
[*] *These authors contributed equally.*
[**] *These authors jointly supervised this work. Correspondence to: t.kouzarides@gurdon.cam.ac.uk; tommaso.leonardi@iit.it; birney@ebi.ac.uk*

October 28, 2021

Supplementary Information

Supplementary text

In silico validations

In order to test the functionality of Nanocompore, we generated an unmodified RNA model from an *in vitro* transcribed (IVT) RNA DRS dataset containing A, U, C and G canonical bases only (from Workman et al). For each 5-mer, we estimated the distribution type and parameters that best fit the observed current intensity and dwell time values. Based on this model, we simulated a reference in silico dataset from a semi-randomly generated unmodified reference sequence (see Materials and Methods). To mimic signal changes induced by RNA modifications we then in silico generated 144 "modified" datasets where the dwell time and signal intensity at defined positions are shifted from the means of the unmodified model by a varying multiple of standard deviations. For each combination of dwell time and signal intensity shift we generated 6 datasets with varying fractions of modified reads (ranging from 10% to 100%) (**Fig. S1A**). We ran Nanocompore on each dataset to test its sensitivity and specificity for identifying these known modified positions. We observed that all the statistical tests implemented in Nanocompore had near perfect precision and recall (0.9889<=AUROC<=0.9947) when the simulated shifts from the model were greater than 2 standard deviations (SD) for both intensity and dwell time and the fraction of modified reads was above 25% (**Fig. S1B** and **Fig. S2**). The GMM method performed better than other methods when only as little as 25% of the reads were modified, although requiring a relatively large shift between the 2 populations (>=2 SD). On the other hand, the KS tests achieved near

Tool	Peak memory (RSS)	Threads	Running Time	I/O (read)	I/O (write)
Minimap2	3.1GB	4	25'	15.7GB	11.2GB
Nanopolish + NanopolishComp	1.8GB	10	50h	2.9TB	2.8TB
Nanocompore	120GB	10	25h	1.6TB	400GB

Table showing the computational requirements of the Nanocompore workflow

perfect precision and recall with milder intensity or dwell time shifts (1 SD) at the expense of the need for a larger population of modified reads. (**Fig. S2**).

Computational requirements

The minimal workflow to run an RNA modification analysis with Nanocompore consists of three steps: mapping (minimap2), signal realignment (nanopolish) and modifications detection (nanocompore). The table reports the computational requirements and execution time of each step for a typical experiment (4 human samples, 2mln reads per sample). The execution time and memory usage are per-sample and the figures reported are the average of six test runs executed at the given number of parallel threads on Intel(R) Xeon(R) E5-2680 v4 CPUs. The execution time varies greatly as a function of several factors, such as CPU model/speed, I/O latency, etc.; therefore, these figures should be considered indicative only.

Supplementary Figures

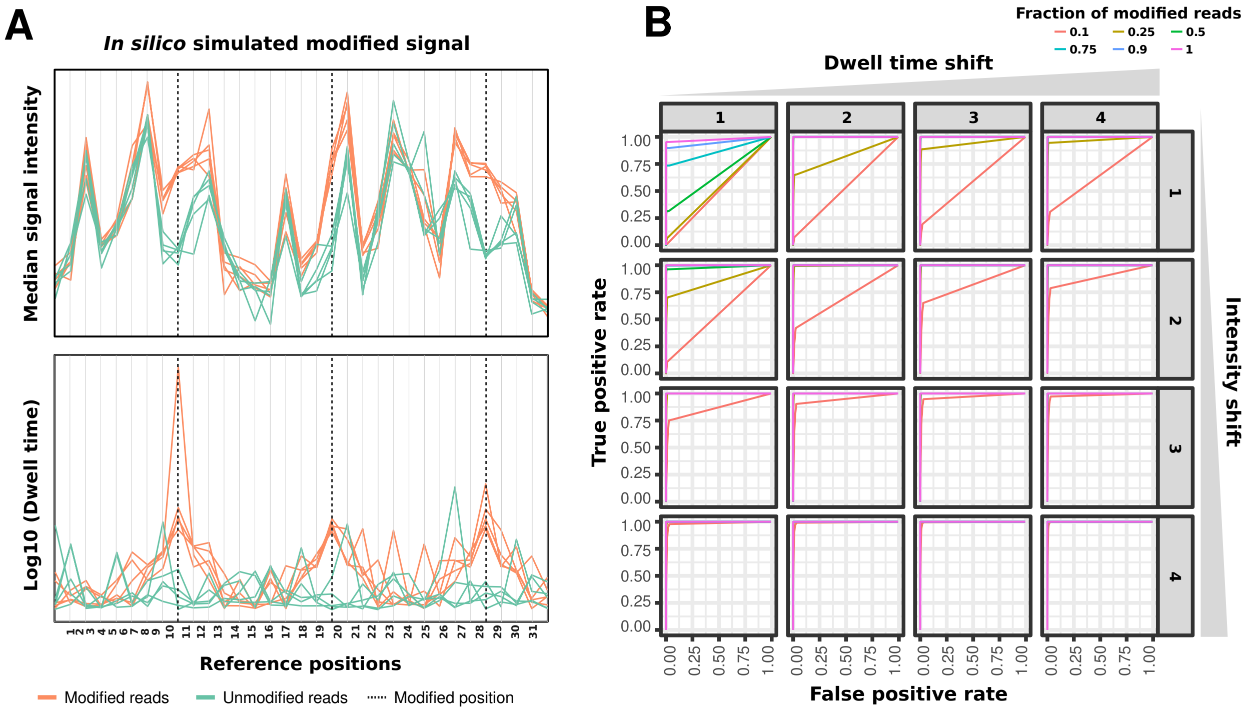

Sup. Fig. 1: *In silico* and *in vitro* validations of Nanocompore. A: Position plots showing the median intensity (top) and dwell time (bottom) for simulated data generated by Nanocompore SimReads. Dashed vertical lines indicate modified positions, where a clear signal shift between unmodified (green) and modified (orange) reads can be seen. Details on sequence generation can be found in the "Simulated reference sequence" of the Materials and Methods. **B:** ROC curves obtained with the GMM method on in silico generated data. The different subplots indicate varying amounts of intensity and dwell time shifts from the unmodified model (from 1 to 4 standard deviations), whereas the different colors indicate varying fractions of modified reads in the generated data (from 10% to 100%). All the comparisons are against a fully unmodified reference dataset.

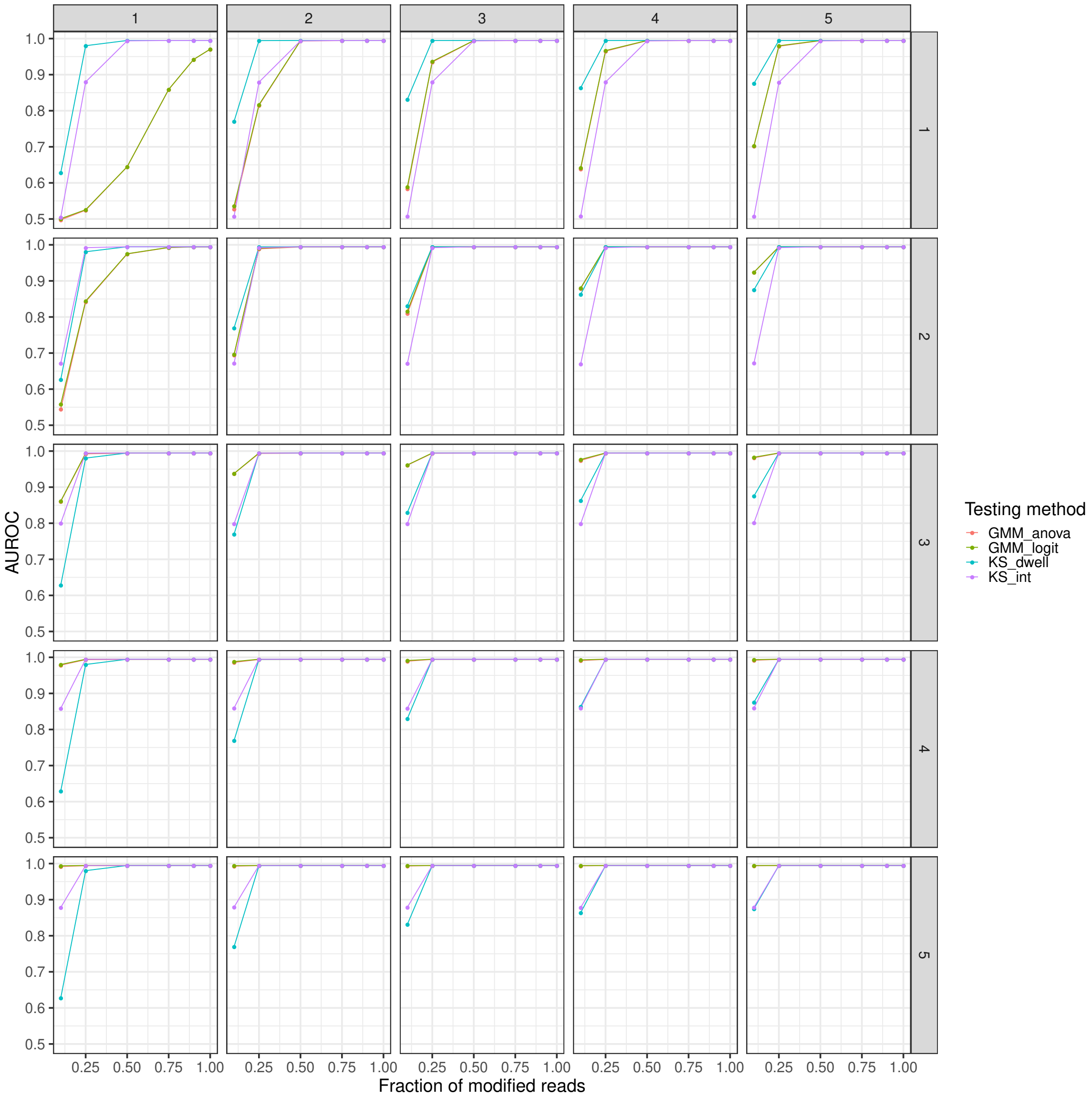

Sup. Fig. 2: Benchmarks on *in silico* data. Plots showing the Area Under the ROC curve (AUROC, y-axis) obtained with Nanocompore on in silico generated data at varying fractions of modified reads (x-axis).

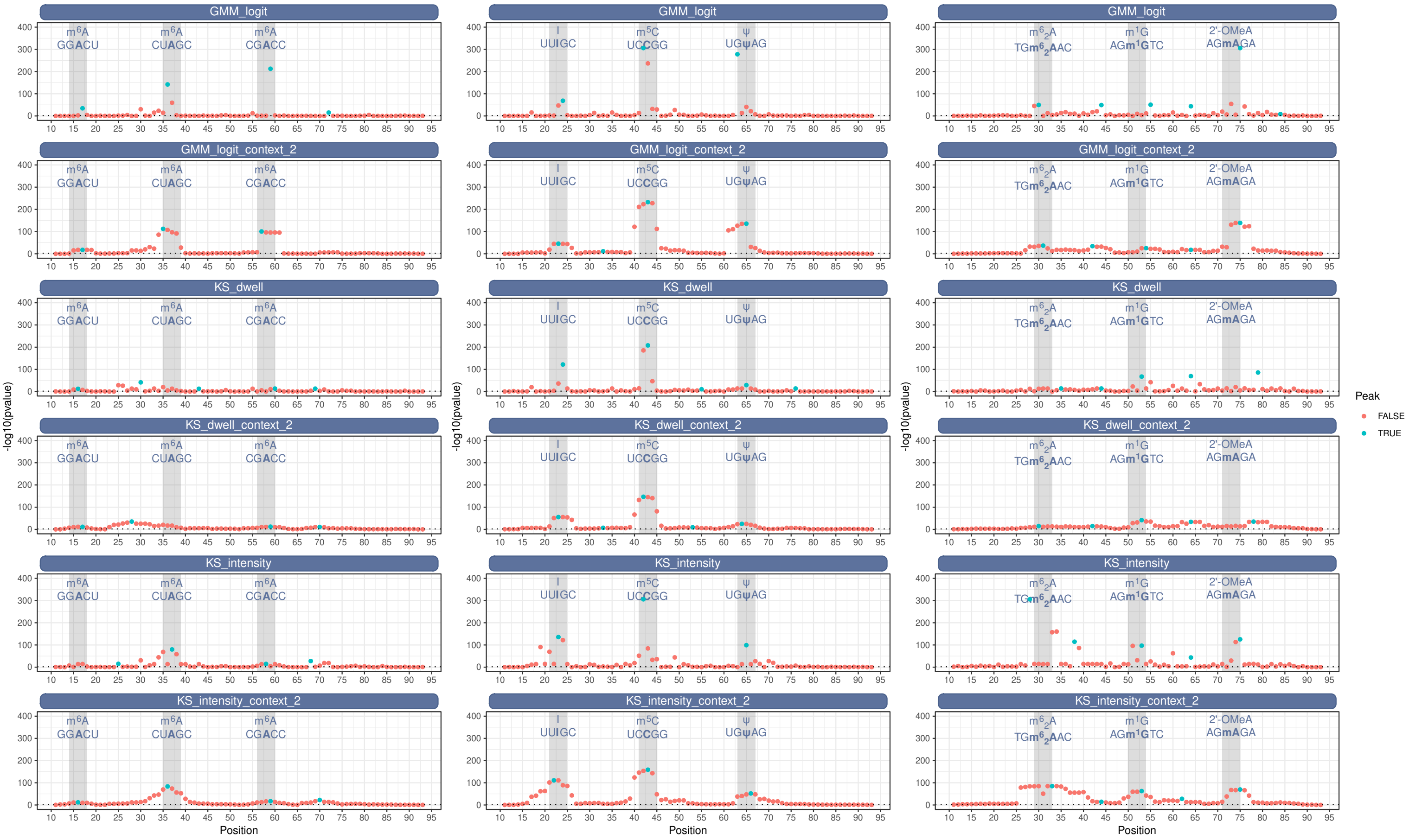

Sup. Fig. 3: Identification of modified sites in synthetic oligos. Nanocompore p-values obtained with the GMM logit, KS intensity and KS dwell time methods, with and without sequence context (y-axis) are reported at each position (x-axis) of Oligo1, Oligo2 and Oligo3. Kmers shown in blue represent the peaks identified through Nanocompore's peak calling procedure. Shaded areas contain the 5 consecutive kmers that contain each modification. The dotted horizontal lines correspond to a p-value of 0.01.

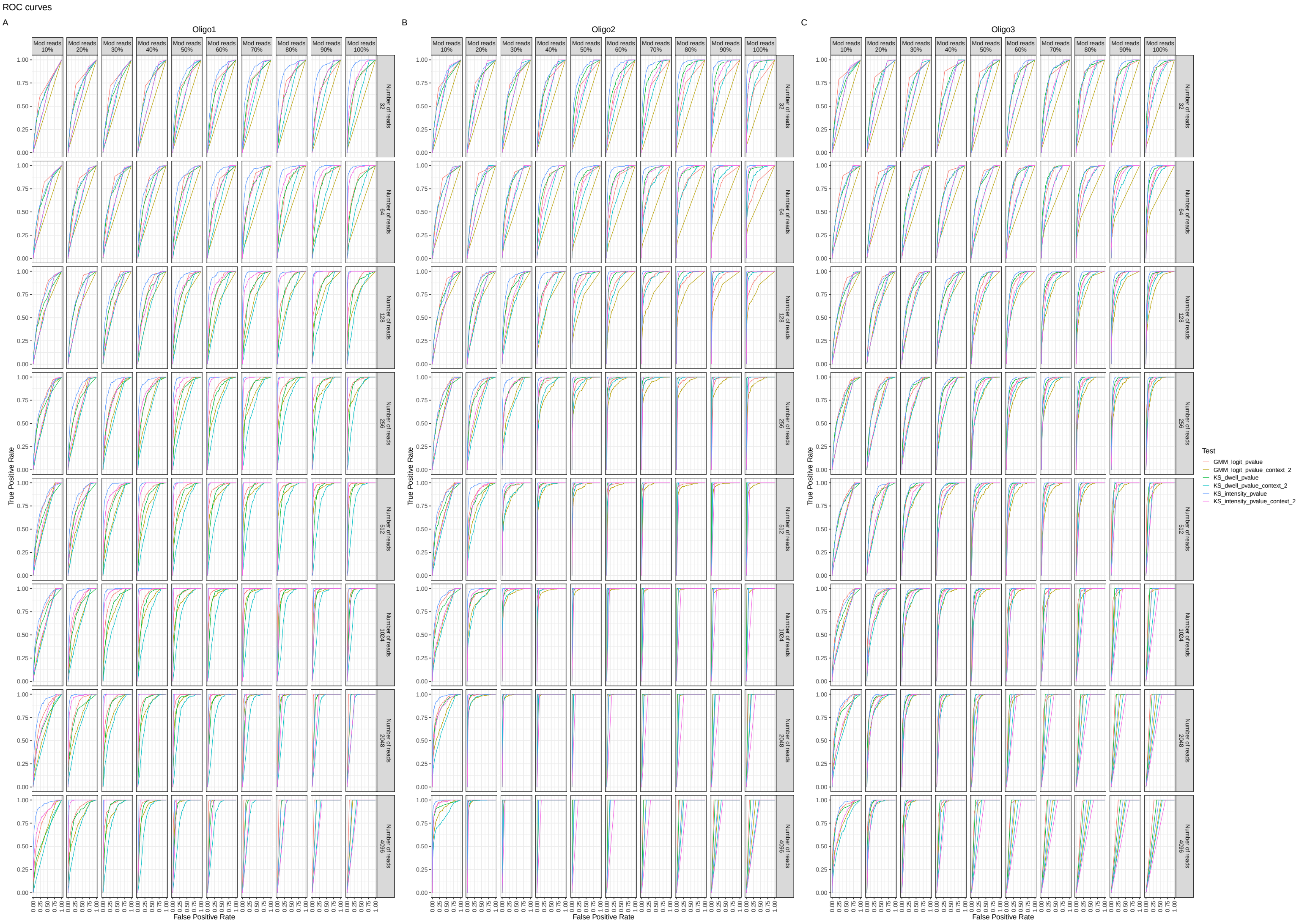

Sup. Fig. 4: Synthetic oligos ROC curves. Plots showing the ROC curves for modification detection in Oligo1 (**A**), Oligo2 (**B**) and Oligo3 (**C**) with the various tests implemented in Nanocompore at varying levels of 1) coverage (rows) and 2) percentage of modified reads (columns). Nominal p-value threshold of 0.05.

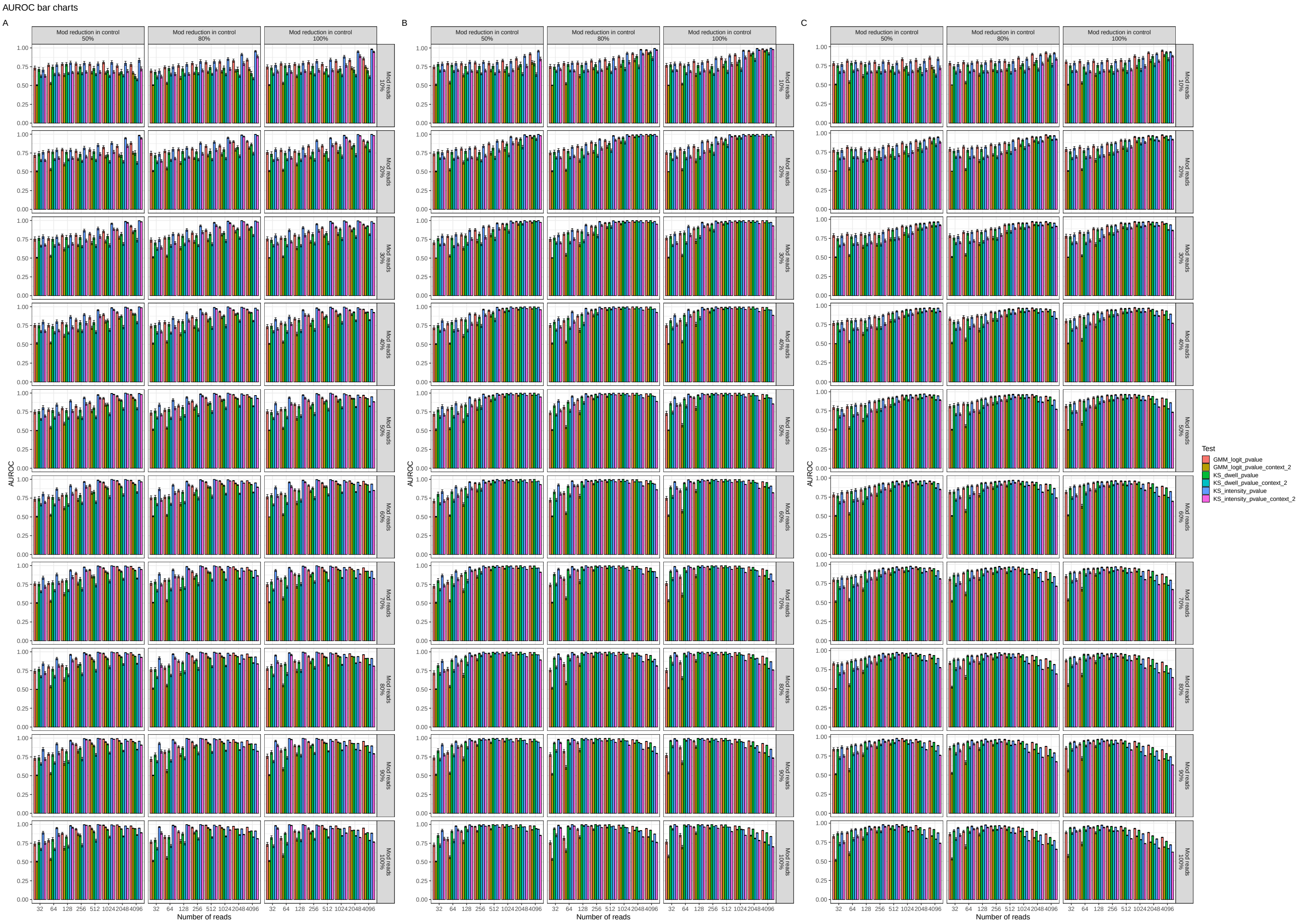

Sup. Fig. 5: Synthetic oligos AUROC plots. Plots showing the area under the ROC curves for modification detection in Oligo1 (**A**), Oligo2 (**B**) and Oligo3 (**C**) with the various tests implemented in Nanocompore at varying levels of 1) coverage (x-axis), 2) percentage of modified reads (row) and 3) modification reduction in control (columns). Nominal p-value threshold of 0.05. The values reported are the means of n=100 artificial samples generated as described (see Materials and Methods). The error bars show the 95% confidence interval.

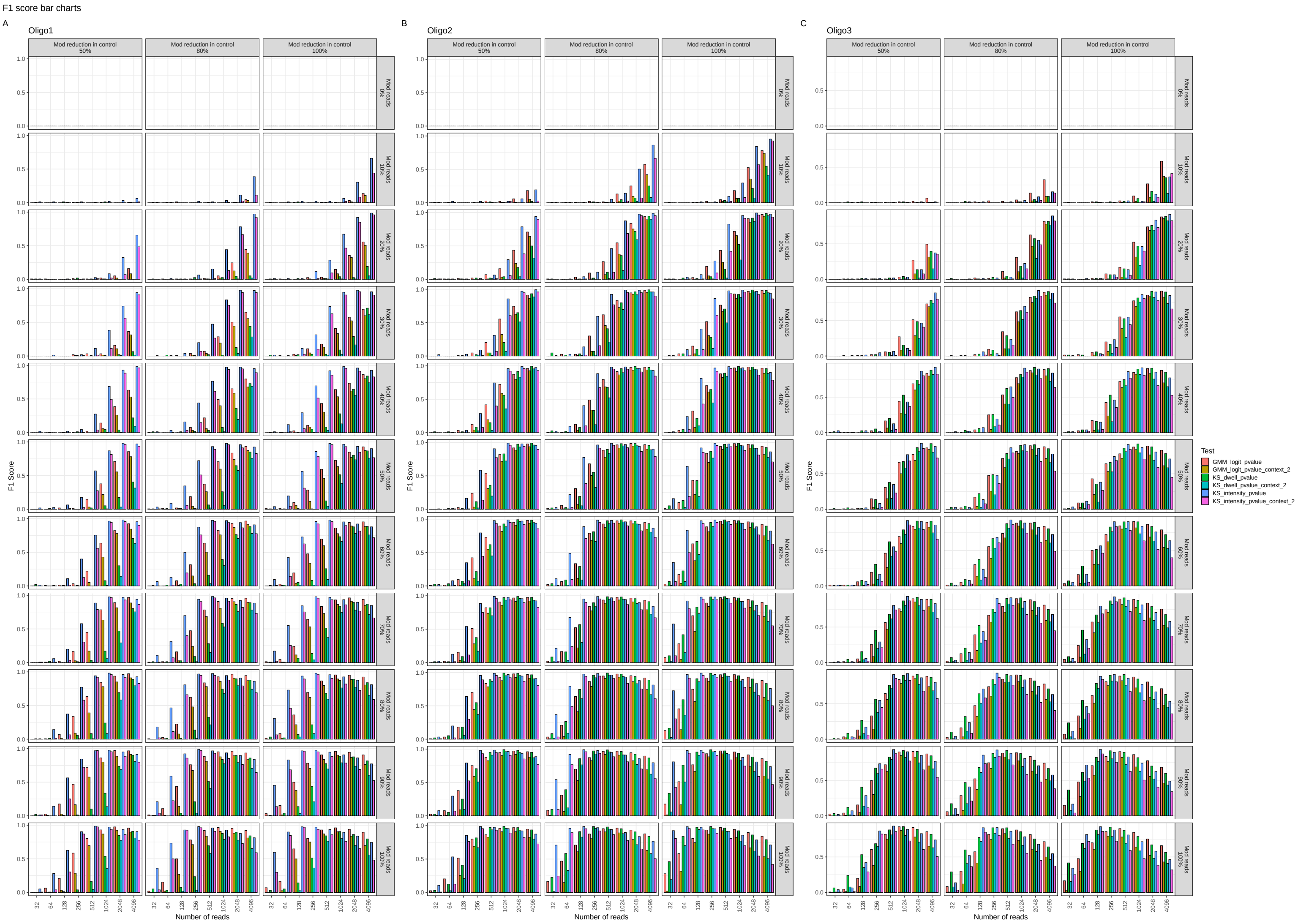

Sup. Fig. 6: Synthetic oligos F1 Score plots. Plots showing the F1 score for modification detection in Oligo1 (**A**), Oligo2 (**B**) and Oligo3 (**C**) with the various tests implemented in Nanocompore at varying levels of 1) coverage (x-axis), 2) percentage of modified reads (row) and 3) modification reduction in control (columns). The values reported are the means of n=100 artificial samples generated as described (see Materials and Methods). The error bars show the 95% confidence interval. TPR and FPR were calculated at a nominal p-value threshold of 0.05.

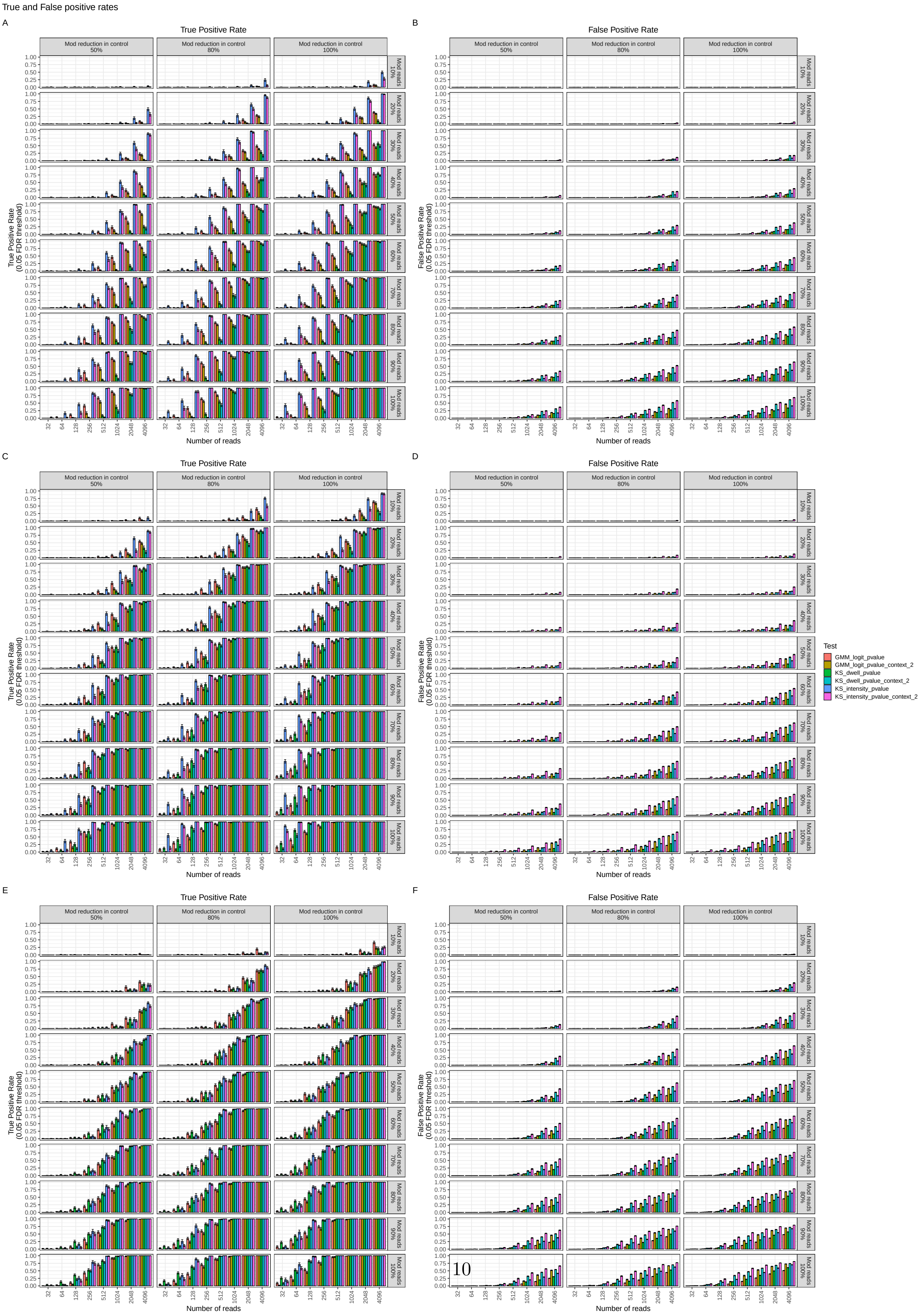

Sup. Fig. 7: legend on next page

Sup. Fig. 7: (previous page) **Synthetic oligos True Positive Rate and False Positive Rate plots.** Plots showing the TPR and FPR for modification detection in Oligo1 (**A,B**), Oligo2 (**C,D**) and Oligo3 (**E,F**) with the various tests implemented in Nanocompore at varying levels of 1) coverage (x-axis), 2) percentage of modified reads (row) and 3) modification reduction in control (columns). The values reported are the means of n=100 artificial samples generated as described (see Materials and Methods). The F1 score was calculated at a nominal p-value threshold of 0.05.

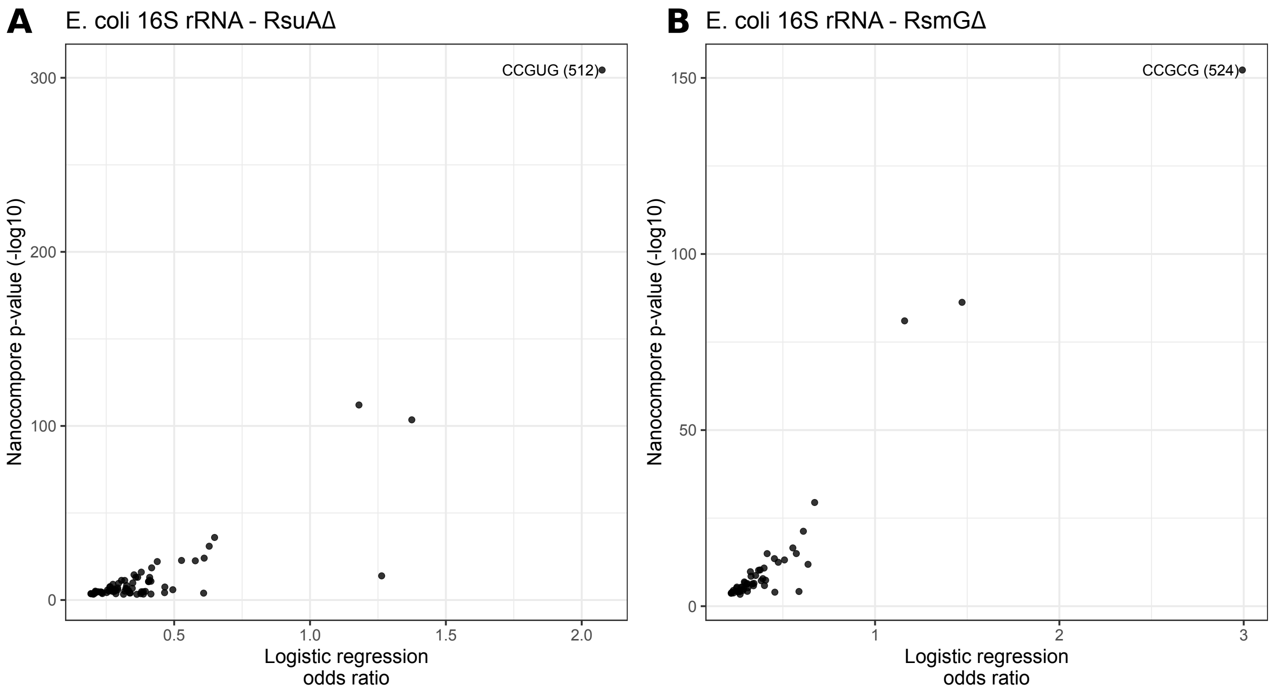

Sup. Fig. 8: Detection of m7G and Ψ in E. coli 16S rRNA. A,B: Sharkfin plots showing results of Nanocompore analyses on 16S rRNA from Escherichia coli strain MRE600 knock-out for RsuA (**A**) and RsmG (**B**). The absolute value of the Nanocompore logistic regression log odd ratio (GMM logit method, x-axis) is plotted against its p-value (-log10, y-axis, see Material and Methods). Each point represents a specific kmer and only kmers corresponding to p-value peaks are shown. Position G527 (i.e. the central G of kmer 524) is a known m7G site while U516 (i.e. the fourth base of kmer 512) is a known Ψ site.

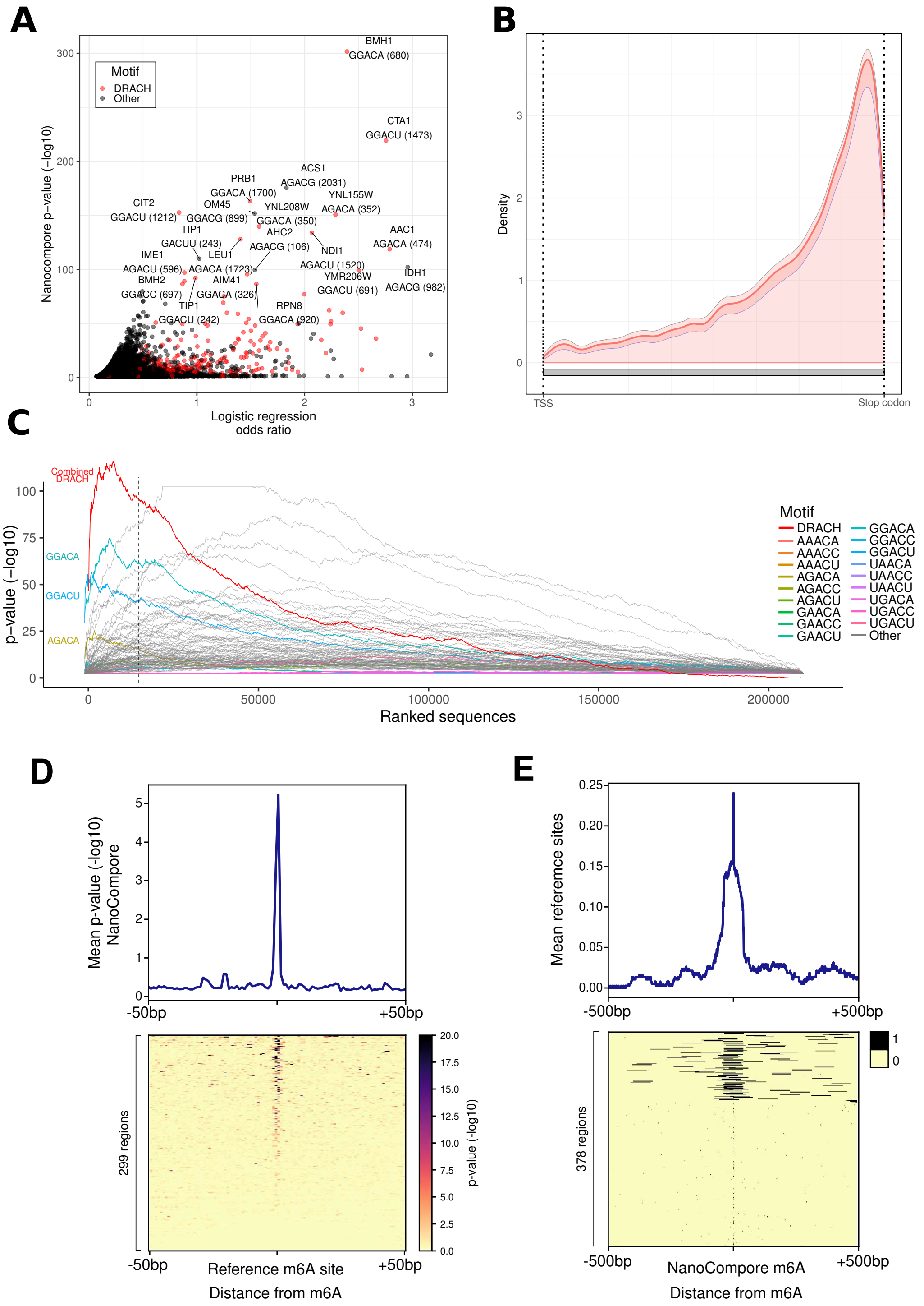

Sup. Fig. 9: Detection of m6A in IME4 KO *S. cerevisiae* **A:**Sharkfin plot showing results of Nanocompore analyses on the transcriptome of IME4 KO vs WT yeast strains. The absolute value of the Nanocompore logistic regression log odd ratio (GMM logit method, x-axis) is plotted against its p-value (-log10, y-axis). Each point represents a specific kmer of a transcript. Red points are DRACH kmers. **B:**Metagene plot showing the distribution of significant m6A sites (GMM logit p-value<0.01 and |log odds ratio|>0.5) identified by Nanocompore (blue). **C:**Sylamer plot showing kmer enrichment in Nanocompore significant sites. The x-axis reports all Nanocompore sites with p-value<0.5 ranked from the most to the least significant. The y-axis reports the uncorrected Sylamer hypergeometric p-value of enrichment (one-sided test) of a certain motif in the first x Nanocompore sites vs the rest. The vertical dotted line delineates Nanocompore sites with p-value<0.01 (to the left of the line). The red line corresponds to the combined p-value (Fisher's method) of all DRACH kmers. **D:**Plot showing the mean Nanocompore p-value across orthogonal reference m6A Sites. **E:**Plot showing the mean coverage in the orthogonal reference m6A data across m6A sites identified by Nanocompore.

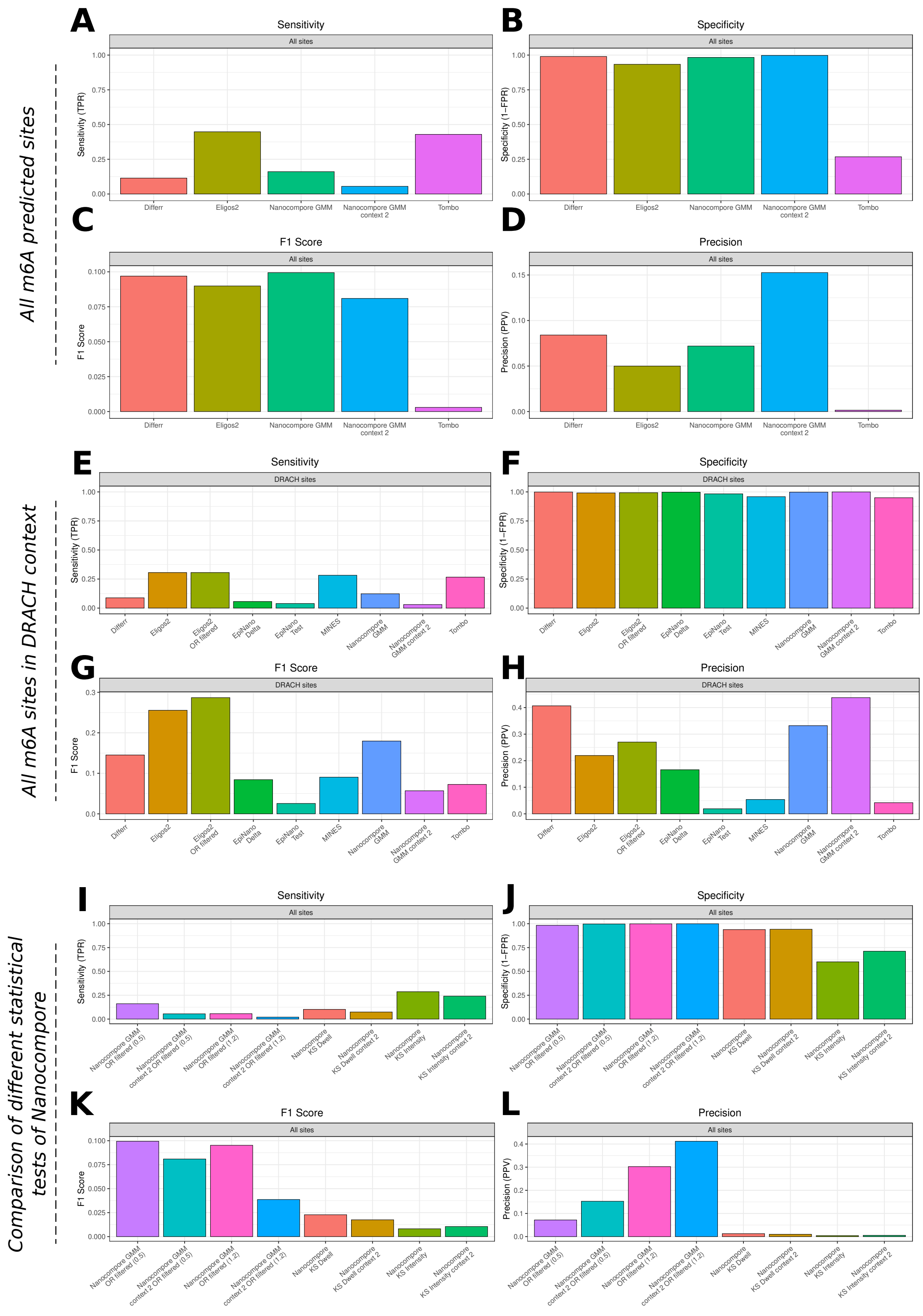

Sup. Fig. 10: Benchmark of Nanocompore against other methods. A-D: Performance metrics of m6A detection in IME4 KO experiment obtained with various methods. The metrics reported are True Positive Rate (**A**), False Positive Rate (**B**), F1 Score (**C**) and Precision (**D**). All metrics were obtained at a nominal adjusted p-value threshold of 0.01. **E-H:**as in **A-D** but the analysis was limited to DRACH kmers. **I-L:**as in **A-D** but the plots only report the metrics for the different statistical tests implemented in Nanocompore.

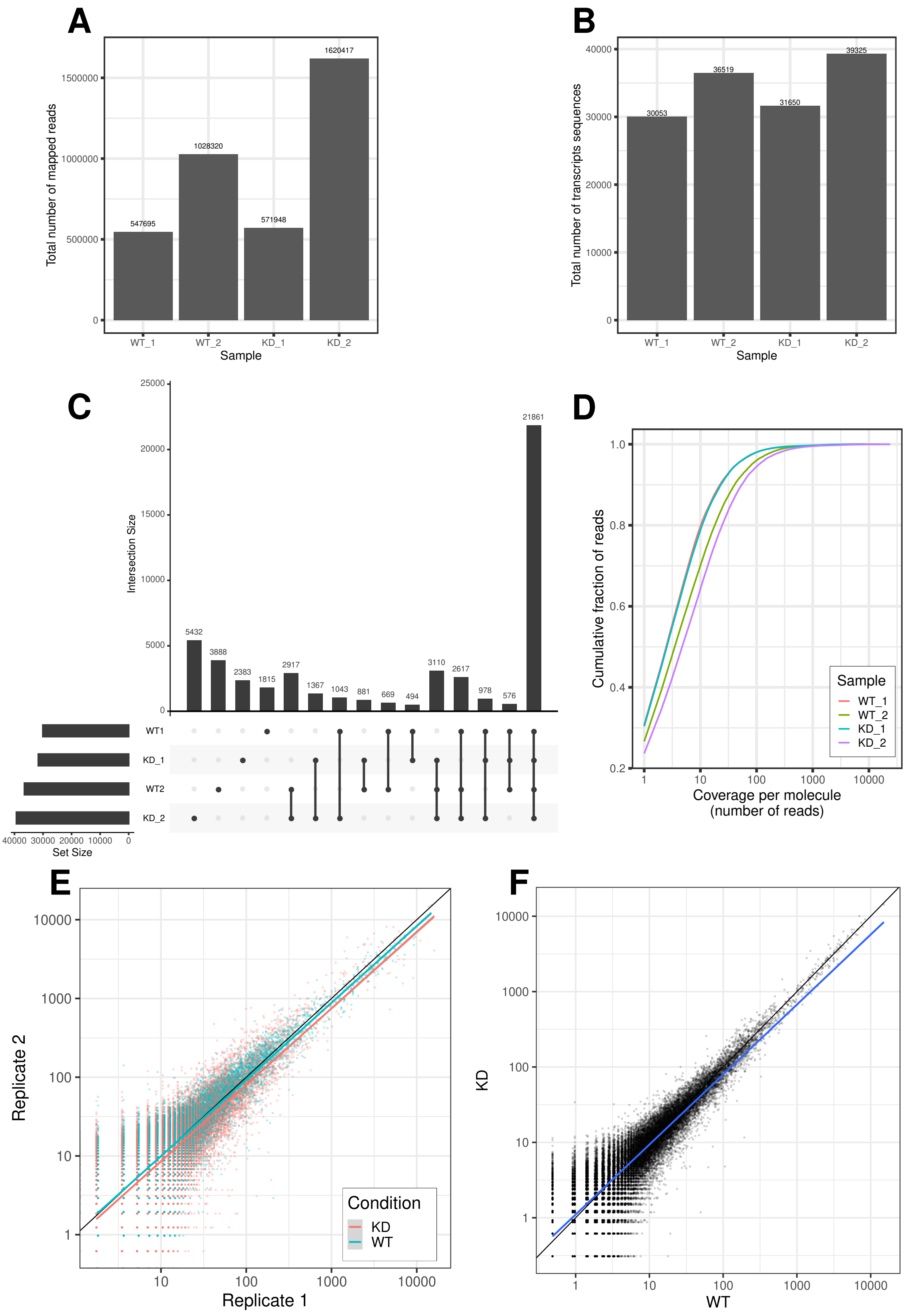

Sup. Fig. 11: legend on next page

Sup. Fig. 11: (previous page) **m6A profiling in the polyA+ transcriptome. A:** Bar chart showing the total number of mapped reads in each sample. Overall total number of reads: 3,768,380 (average 942,095 per sample). **B:** Bar chart and intersections plots showing the total number of sequences transcripts in each sample. Average: 34,386.75 distinct transcripts per sample **C:** Bar chart and intersections plots showing the total number of sequences transcripts in each sample and in every combination of samples. **D:** Cumulative fraction of reads mapping to transcripts with increasing degree of total coverage. For example, the point (10,0.6) on the purple line indicates that in the KD2 sample ~60% of the reads map to transcripts with coverage of 10x or lower. **E:** Scatter plot showing the correlation in transcript abundance between replicates. R^2 of 0.757 and 0.937 for WT and KD respectively. **F:** Scatter plot showing the correlation in transcript abundance between WT and KD after averaging replicates. R^2=0.969.

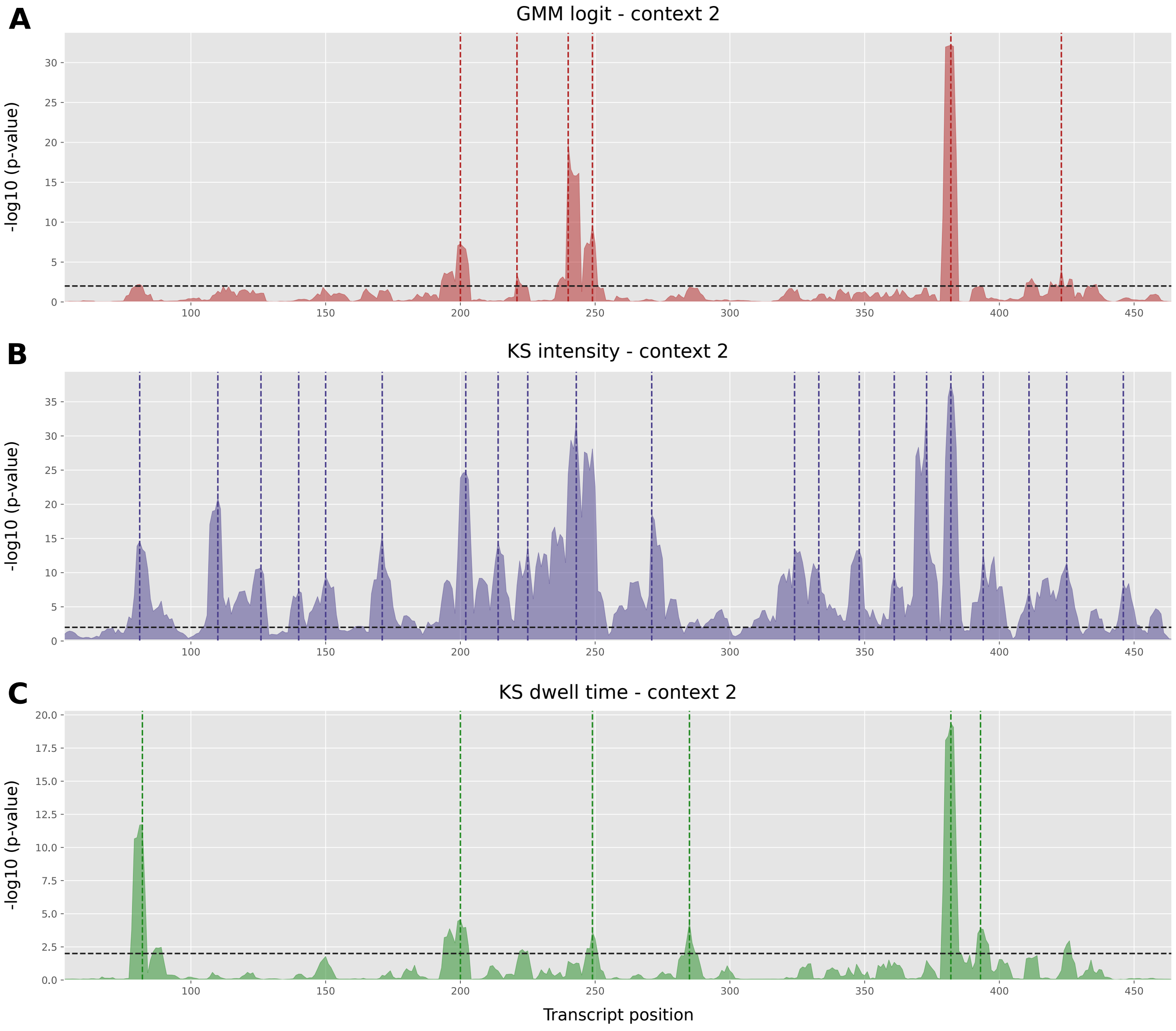

Sup. Fig. 12: m6A peak calling Example of peak calling in the -log10 p-value space for transcript ENST00000501597 (human RPL41 gene), for the following methods using a context of 2 bases: GMM-logit method (**A**, red), KS test intensity (**B**, blue) and KS test Dwell time (**C**, green). The horizontal black lines represent the p-value threshold (0.01) and vertical dotted lines indicate the position of peaks called for each condition. The median coverage of this transcript was 948.5 for WT and 1700.0 for KD (average of two replicates).

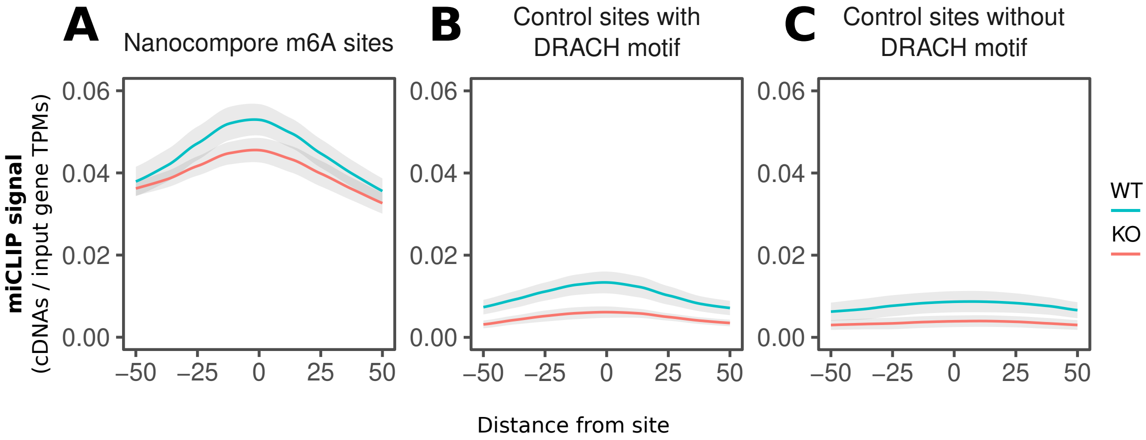

Sup. Fig. 13: m6A miCLIP coverage of Nanocompore sites Plot showing the m6A miCLIP signal enrichment in a 100nt window around m6A sites identified by Nanocompore (**A**, GMM logit context 2 p-value<0.01), control sites with DRACH motif that were not identified by Nanocompore (**B**, number of sites: 1948) and control sites without DRACH motif that were not identified by Nanocompore (**C**, number of sites: 1207). The y-axis shows the mean input-normalised miCLIP counts across sites. Shaded regions on the plot represent the mean +/- the standard deviation at each position in the profile (WT miCLIP n=4, KO n=2). Both the mean and bounds were smoothed using loess regression with a span of 0.6.

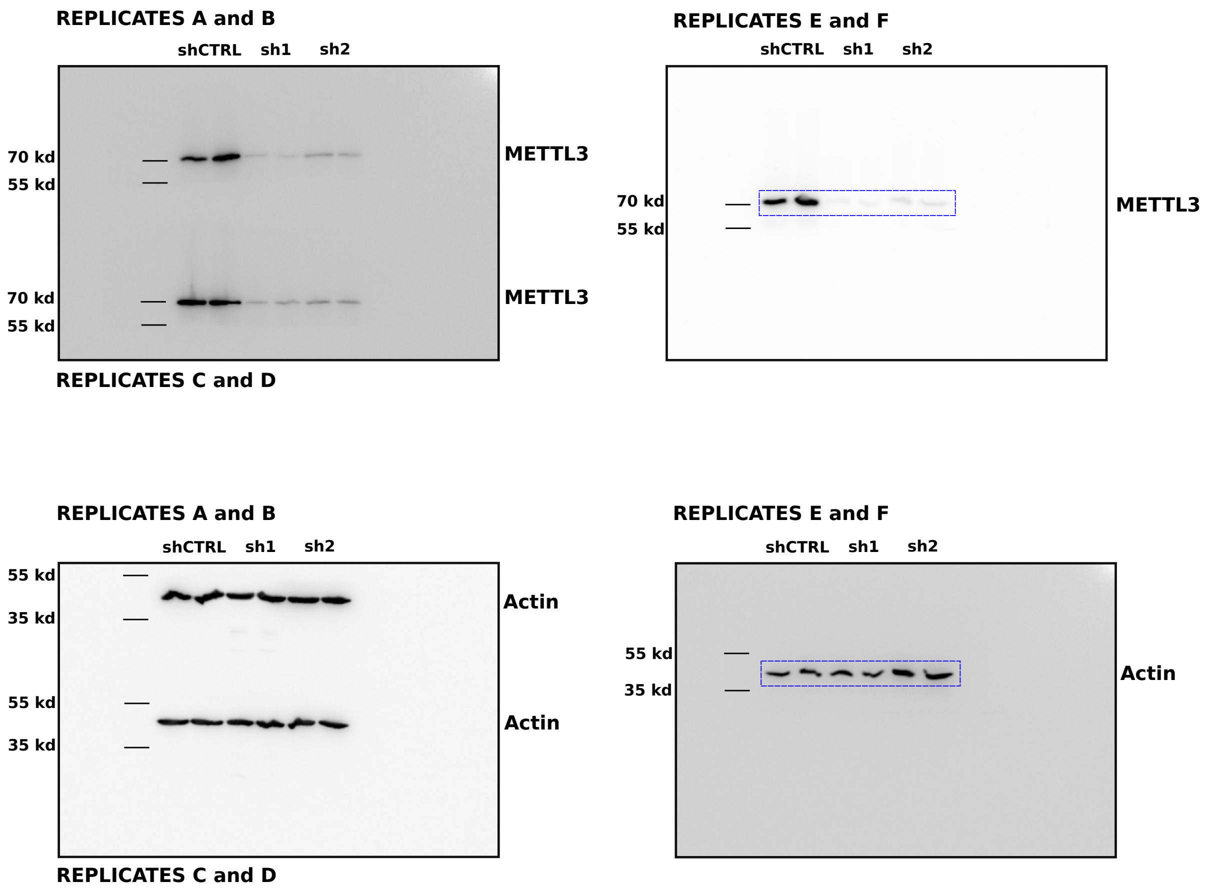

Sup. Fig. 14: Western Blot showing METTL3 reduction upon KD. Full uncropped scans of Western Blots showing a reduction of METTL3 protein levels in the 6 replicate KD experiments (labeled A-F) used for validating by qPCR the presence of m6A in 7SK.

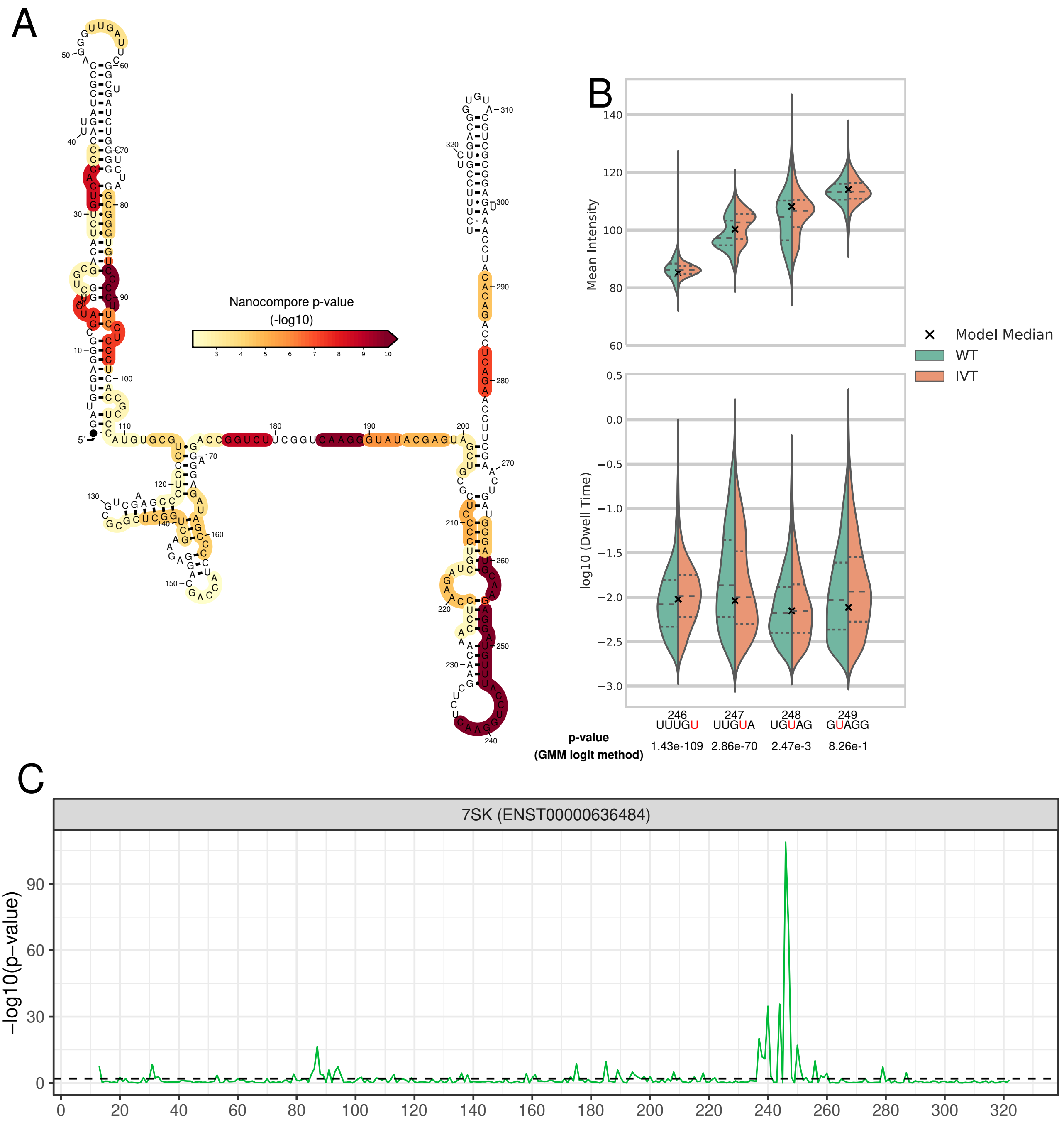

Sup. Fig. 15: Modification profile of 7SK from the analysis on an IVT sample. A: Secondary structure of 7SK with the Nanocompore IVT vs WT p-value (GMM-logit) overlaid as a color scale. For each nucleotide the color indicates the lowest p-value among those of the 5 kmers that overlap it. Only p-values<0.01 are shown in color. **B:** Violin plots showing the distributions of median intensity (top) and scaled $\log_{10}$ dwell time (bottom) for kmers encompassing the known pseudouridine site U250. the Hexim1 binding sites and neighbouring kmers. **C:** RNA modification profile of 7SK, showing the Nanocompore GMM-logit p-value (y axis, -$\log_{10}$) across the transcript length. All coordinates refer to the first nucleotide of each kmer relative to ENST00000636484.

Supplementary Tables

	Detection strategy	Statistical Framwork used	Modifications supported	Restricted to sequence	Base resolution	Read resolution	Package manager	Versioned	Actively updated and maintained
Epinano	Alternative base model of basecalling 'errors'	Support vector machine classifier	Only m6A model available	Yes, kmers containing a single A only	Single base	Consensus	No	Yes	Yes
Tombo	Signal level comparision of 2 conditions for current intensity or alternative intensity model	Pairwise statistical tests (KS, U-test, t-test)	m5C with the modelPotentially any modifications with comparative strategy	No	5 bases window	Single read for m5CConsensus for other modifications	pip / conda	Yes	No
MINES	Alternative intensity model from tombo + model based on CLIP-Seq motifs	Support vector machine classifier	Only m6A model available	Yes, only 4 kmers from DRACH motif	5 base window	Consensus isoform	No	No	No
Nanocompore	Signal level comparision of 2 conditions for both current intensity and dwell time	Pairwise statistical tests (KS on intensity and dwell time) + Gaussian mixture model coupled with logistic regression or Anova	Potentially any modifications	No	5 base window	Consensus and single read	pip / conda / poetry	Yes	Yes
Eligos	Basecall level comparison of mismatch errors compared with matching unmodified RNA, cDNA sequences or provded background error model	G statistics	Potentially any modifications	No	5 base window	Consensus	docker	No	Yes
differr	Basecall level comparison of mismatch errors beetween 2 conditions	Chi-Square and Odd ratio of mismatch	Potentially any modifications	No	5 base window	Consensus	No	No	No

Table S 1: Feature comparison of Nanopore-based modification detection software.

Construct name	sg- / shRNA sequence
METTL3 gRNA1	GCTCAACATACCCGTACTAC
METTL3 gRNA2	CTGTTGTGATATCCGCTACC
METTL3 sh1	CGTCAGTATCTTGGGCAAGTTCTCGAGAACTTGCCCAAGATACTGACGTTTTTG
METTL3 sh2	GCTGCACTTCAGACGAATTATCTCGAGATAATTCGTCTGAAGTGCAGCTTTTTG
METTL3 Scramble	CAACAAGATGAAGAGCACCAACTCGAGTTGGTGCTCTTCATCTTGTTGTTTTTG

Table S 2: Sequences of sgRNAs and shRNAs used for KD and KO experiments.

Oligo name	Oligo sequence (T7 promoter in bold)
T7 7SK	5'-**TATTAGTACTTAATACGACTCACTATAG**GGATGT<br>GAGGGCGATCTGGCTGCGACATCTGTCACCCCATTGA<br>TCGCCAGGGTTGATTCGGCTGATCTGGCTGGCTAGGC<br>GGGTGTCCCCTTCCTCCCTCACCGCTCCATGTGCGTC<br>CCTCCCGAAGCTGCGCGCTCGGTCGAAGAGGACGACC<br>ATCCCCGATAGAGGAGGACCGGTCTTCGGTCAAGGGT<br>ATACGAGTAGCTGCGCTCCCCTGCTAGAACCTCCAAA<br>CAAGCTCTCAAGGTCCATTTGTAGGAGAACGTAGGGT<br>AGTCAAGCTTCCAAGACTCCAGACACATCCAAATGAG<br>GCGCTGCATGTGGCAGTCTGCCTTTCTTT-3'
T7 7SK Reverse Complement	5'-AAAGAAAGGCAGACTGCCACATGCAGCGCCTCAT<br>TTGGATGTGTCTGGAGTCTTGGAAGCTTGACTACCCT<br>ACGTTCTCCTACAAATGGACCTTGAGAGCTTGTTTGG<br>AGGTTCTAGCAGGGGAGCGCAGCTACTCGTATACCCT<br>TGACCGAAGACCGGTCCTCCTCTATCGGGGATGGTCG<br>TCCTCTTCGACCGAGCGCGCAGCTTCGGGAGGGACGC<br>ACATGGAGCGGTGAGGGAGGAAGGGGACACCCGCCTA<br>GCCAGCCAGATCAGCCGAATCAACCCTGGCGATCAAT<br>GGGGTGACAGATGTCGCAGCCAGATCGCCCTCACATC<br>**CCTATAGTGAGTCGTATTAAGTACTAATA**-3'

Table S 3: DNA oligo sequences used to produce double stranded DNA template for 7SK in vitro transcription.

Oligo name	Oligo sequence
L3-ATT	5'Phos - WNATTAGATCGGAAGAGCGGTTCAG - 3'Bio
L3-AGG	5'Phos - WNAGGAGATCGGAAGAGCGGTTCAG - 3'Bio
L3-TTA	5'Phos - WNTTAAGATCGGAAGAGCGGTTCAG - 3'Bio
L3-TGC	5'Phos - WNTGCAGATCGGAAGAGCGGTTCAG - 3'Bio

Table S 4: Sequence of miCLIP barcoded adapters.

Full name	Gene Symbol	Ensembl gene ID	Ensembl Transcript ID	Targeting sequence
RNA component of 7SK nuclear ribonucleoprotein	RN7SK	ENSG00000283293	ENST00000636484.1	5'-AAAGAAAGGCAGACTGCCAC-3'
Ribonuclease P RNA component H1	RPPH1	ENSG00000277209	ENST00000516869.1	5'-AATGGGCGGAGGAGAGTAGT-3'
RNA U2 small nuclear 1	RNU2-1	ENSG00000274585	ENST00000618664.1	5'-TGGTGCACCGTTCCTGGAGG-3'
RNA component of mitochondrial RNA processing endoribonuclease	RMRP	ENSG00000277027	ENST00000602361.1	5'-ACAGCCGCGCTGAGAATGAG-3'

Table S 5: Targeted ncRNAs

	Median occurrences	Mean occurrences	Min occurrences	Max occurrences	kmers found	kmers expected	Percent kmers found
5-mers	970	968	695	1023	1024	1024	100
6-mers	242	241	214	275	4092	4092	100
7-mers	60	60	45	89	16356	16356	100
8-mers	15	15	8	26	65376	65376	100
9-mers	4	3	0	9	261268	261312	99.98
10-mers	1	1	0	6	706080	1044480	67.6
11-mers	1	1	0	4	913965	4174848	21.89

Table S 6: Coverage of kmers (5 to 11 mers) found in the in silico generated reference sequences. The sequence set was designed to optimise the 9-mers content. The expected kmer count excludes the sequences containing homopolymers longer than 5 bases which were intentionally avoided when designing the reference sequence.
