# Supplementary material for: RNA modifications detection by comparative Nanopore direct RNA sequencing
Source: Nat Commun. 2021 Dec 10;12:7198. doi: 10.1038/s41467-021-27393-3 (PMC8664944; doi:10.1038/s41467-021-27393-3)
Supplement: Supplementary file 2 — Reporting Summary [file 41467_2021_27393_MOESM2_ESM.pdf]

nature portfolio

Corresponding author(s): Tony Kouzarides
Tommaso Leonardi
Ewan Birney

Last updated by author(s): 2021/10/27

Reporting Summary

Nature Portfolio wishes to improve the reproducibility of the work that we publish. This form provides structure for consistency and transparency in reporting. For further information on Nature Portfolio policies, see our Editorial Policies and the Editorial Policy Checklist.

Statistics

For all statistical analyses, confirm that the following items are present in the figure legend, table legend, main text, or Methods section.

n/a	Confirmed	
☐	☒	The exact sample size (*n*) for each experimental group/condition, given as a discrete number and unit of measurement
☐	☒	A statement on whether measurements were taken from distinct samples or whether the same sample was measured repeatedly
☐	☒	The statistical test(s) used AND whether they are one- or two-sided<br>*Only common tests should be described solely by name; describe more complex techniques in the Methods section.*
☐	☒	A description of all covariates tested
☐	☒	A description of any assumptions or corrections, such as tests of normality and adjustment for multiple comparisons
☐	☒	A full description of the statistical parameters including central tendency (e.g. means) or other basic estimates (e.g. regression coefficient) AND variation (e.g. standard deviation) or associated estimates of uncertainty (e.g. confidence intervals)
☐	☒	For null hypothesis testing, the test statistic (e.g. *F*, *t*, *r*) with confidence intervals, effect sizes, degrees of freedom and *P* value noted<br>*Give P values as exact values whenever suitable.*
☒	☐	For Bayesian analysis, information on the choice of priors and Markov chain Monte Carlo settings
☒	☐	For hierarchical and complex designs, identification of the appropriate level for tests and full reporting of outcomes
☐	☒	Estimates of effect sizes (e.g. Cohen's *d*, Pearson's *r*), indicating how they were calculated

Our web collection on statistics for biologists contains articles on many of the points above.

Software and code

Policy information about availability of computer code

Data collection	Fast5 files for Nanopore direct RNA sequencing experiments were converted to CRAM format with ONT2CRAM (https://github.com/EGA-archive/ont2cram). The resulting CRAM files were uploaded to ENA under accession numbers PRJEB44511 and PRJEB35148. Fastq files for miCLIP experiments were uploaded to ENA under accession number PRJEB35148. A detailed description of the procedures for data collection and generation is available in the Meterials and Methods section of the paper.
Data analysis	Custom code used in this paper is deposited on Github in the following repositories:<br>github.com/tleonardi/nanocompore<br>github.com/a-slide/MetaCompore<br>github.com/tleonardi/nanocompore_paper_analysis<br>github.com/tleonardi/nanocompore_pipeline<br><br>The analysis done in the paper made user of the following software packages:<br>Minimap2 v2.14 (for unmodified model generation)<br>Minimap2 v2.16 (for experimental dRNASeq datasets)<br>Nanopolish v0.10.1<br>Nanopolish Eventalign_collapse v0.5<br>Nanocompore v1.0.0rc3<br>RNAfold 2.4.15<br>Guppy v3.2.10 (for synthetic oligos)<br>Guppy v3.1.5 (for experimental dRNASeq samples)<br>pycoQC v2.2.4<br>Bedparse v0.2.2<br>samtools v1.9<br>Guitar v2.8.0

R/Bioconductor v3.13
Sylamer v12-342
R2R v1.0.5
STAR v2.4.0.1
Bedtools v2.28.0
deeptools v3.3.0
Metacompore v0.1.2
Epinano v1.2.0
Eligos v2.0.0
Tombo v1.5.1
differr commit 7da0652
mines commit 737d16c
Nanocompore v1.0.3 (for benchmarks against other methods)
Minimap2 v2.17 (for benchmarks against other methods)
pyBioTools v0.2.7
f5c v0.6

For manuscripts utilizing custom algorithms or software that are central to the research but not yet described in published literature, software must be made available to editors and reviewers. We strongly encourage code deposition in a community repository (e.g. GitHub). See the Nature Portfolio guidelines for submitting code & software for further information.

Data

Policy information about availability of data

All manuscripts must include a data availability statement. This statement should provide the following information, where applicable:

- Accession codes, unique identifiers, or web links for publicly available datasets
- A description of any restrictions on data availability
- For clinical datasets or third party data, please ensure that the statement adheres to our policy

The Nanopore direct RNA sequencing and miCLIP datasets generated in this study have been deposited in the European Nucleotide Archive database under accession codes PRJEB44511 and PRJEB 35148.

Field-specific reporting

Please select the one below that is the best fit for your research. If you are not sure, read the appropriate sections before making your selection.

☒ Life sciences ☐ Behavioural & social sciences ☐ Ecological, evolutionary & environmental sciences

For a reference copy of the document with all sections, see nature.com/documents/nr-reporting-summary-flat.pdf

Life sciences study design

All studies must disclose on these points even when the disclosure is negative.

Sample size	The sample size for RNA sequencing experiments was not predetermined with power analyses, but each experiments was done in biological duplicate (for human samples) or triplicate (for yeast samples). Sequencing experiments for RNAs generated in vitro (i.e. synthetic oligos and IVT samples) were not replicated.
Data exclusions	Low quality Nanopore reads were discarded using default pass/fail parameters of Guppy. For sequencing datatasets of synthetic oligonucleotides, reads shorter than 100nt were discarded as described in detail in the Materials and Methods section.
Replication	At least two replicates per biological sample were sequenced and no samples were excluded from analyses.
Randomization	Samples were not randomized but all sequencing experiments were done by treating all samples in parallel.
Blinding	Sequencing experiments were done without blinding, but all samples were treated in parallel.

Reporting for specific materials, systems and methods

We require information from authors about some types of materials, experimental systems and methods used in many studies. Here, indicate whether each material, system or method listed is relevant to your study. If you are not sure if a list item applies to your research, read the appropriate section before selecting a response.

Materials & experimental systems

n/a	Involved in the study
☐	☒ Antibodies
☐	☒ Eukaryotic cell lines
☒	☐ Palaeontology and archaeology
☒	☐ Animals and other organisms
☒	☐ Human research participants
☒	☐ Clinical data
☒	☐ Dual use research of concern

Methods

n/a	Involved in the study
☒	☐ ChIP-seq
☒	☐ Flow cytometry
☒	☐ MRI-based neuroimaging

Antibodies

Antibodies used	Anti N6-methyladenosine antibody (Abcam, ab151230, lot GR3319501-1) and anti-GFP antibody as negative control (Abcam, ab290, lot GR3321575-1). Anti METTL3 antibody (Abcam ab195352, lot GR3247121) and anti beta-Actin as loading control (Abcam, ab8227, lot GR3255609-1).
Validation	Anti m6A antibody validated by the manufacturer by Nucleotide Array and tested in human, mouse and Drosophila Melanogaster. Anti-GFP antibody extensively validated by the manufacturer (species independent). Anti-METTL3 antibody validated by the manufacturer by Knock Out; reacts with Mouse, Human and Rat METTL3. Anti beta-Actin antibody extensively validated by the manufacturer.

Eukaryotic cell lines

Policy information about cell lines

Cell line source(s)	MOLM13 cells were obtained from the Sanger Institute Cancer Cell collection.
Authentication	Cell lines were not authenticated
Mycoplasma contamination	Cell lines were regularly tested for mycoplasma contamination and shown to be negative.
Commonly misidentified lines (See ICLAC register)	MOLM13 cells are not listed in the ICLAC database v11.
